# Skeletal gene expression in the temporal region of the reptilian embryos: implications for the evolution of reptilian skull morphology

**DOI:** 10.1186/2193-1801-2-336

**Published:** 2013-07-23

**Authors:** Masayoshi Tokita, Win Chaeychomsri, Jindawan Siruntawineti

**Affiliations:** Graduate School of Life and Environmental Sciences, University of Tsukuba, Tenno-dai 1-1-1, Tsukuba, Ibaraki, 305-8572 Japan; Department of Zoology, Kasetsart University, 50 Ngam Wong Wan Road, Chatuchak, Bangkok, 10900 Thailand; Department of Organismic and Evolutionary Biology, Harvard University, 16 Divinity Avenue, Cambridge, MA 02138 USA

**Keywords:** Reptiles, Skull, Morphology, Development, Osteogenesis, Heterotopy

## Abstract

Reptiles have achieved highly diverse morphological and physiological traits that allow them to exploit various ecological niches and resources. Morphology of the temporal region of the reptilian skull is highly diverse and historically it has been treated as an important character for classifying reptiles and has helped us understand the ecology and physiology of each species. However, the developmental mechanism that generates diversity of reptilian skull morphology is poorly understood. We reveal a potential developmental basis that generates morphological diversity in the temporal region of the reptilian skull by performing a comparative analysis of gene expression in the embryos of reptile species with different skull morphology. By investigating genes known to regulate early osteoblast development, we find dorsoventrally broadened unique expression of the early osteoblast marker, *Runx2*, in the temporal region of the head of turtle embryos that do not form temporal fenestrae. We also observe that *Msx2* is also uniquely expressed in the mesenchymal cells distributed at the temporal region of the head of turtle embryos. Furthermore, through comparison of gene expression pattern in the embryos of turtle, crocodile, and snake species, we find a possible correlation between the spatial patterns of *Runx2* and *Msx2* expression in cranial mesenchymal cells and skull morphology of each reptilian lineage. Regulatory modifications of *Runx2* and *Msx2* expression in osteogenic mesenchymal precursor cells are likely involved in generating morphological diversity in the temporal region of the reptilian skull.

**Electronic supplementary material:**

The online version of this article (doi:10.1186/2193-1801-2-336) contains supplementary material, which is available to authorized users.

## Introduction

Amniotes (Amniota) consist of two large groups of tetrapod vertebrates, Synapsida and Reptilia, that diverged from one another over 300 million years ago (Ma) (Carroll, 1988;Modesto & Anderson,, Modesto & Anderson, Modesto & Anderson,^2004^, Benton, 2005). The synapsids are represented today by mammals while reptiles by extant turtles, tuatara, lizards, snakes, crocodiles, birds, and their extinct relatives, including dinosaurs and pterosaurs. Over time, reptiles have evolved highly diverse morphological and physiological traits that allow them to exploit various ecological niches and resources on the land, in water, and in the air.

**Figure 1:**
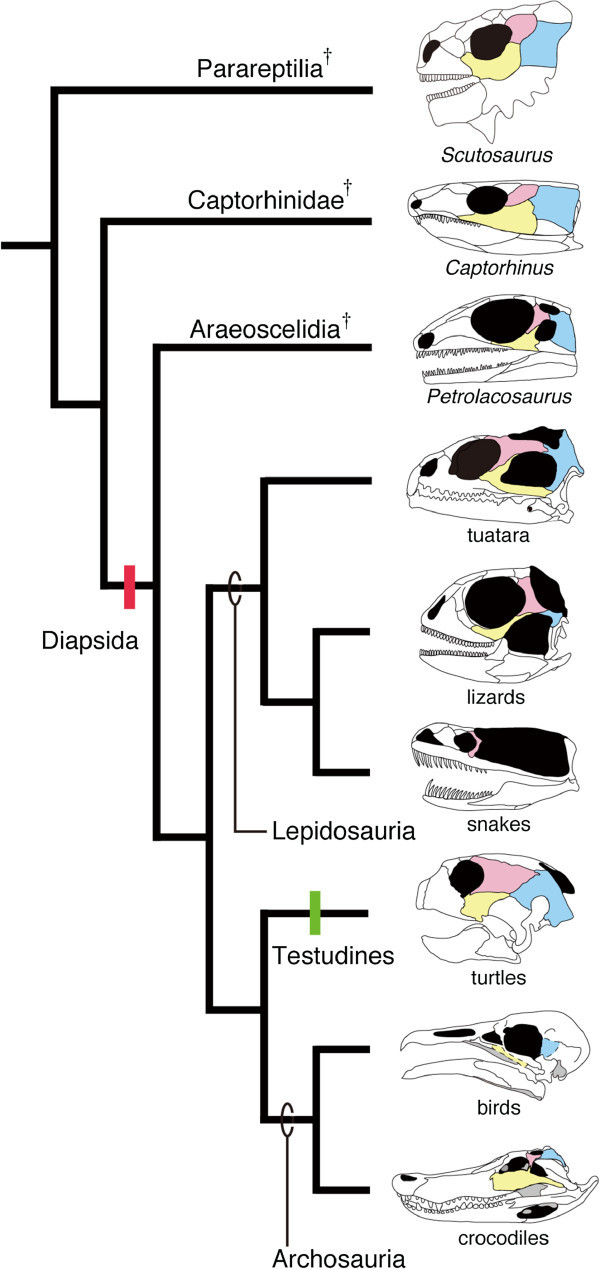
**Simplified phylogeny of the Reptilia highlighting diversity of their skull morphology**. Paleontological evidence suggests that all reptiles, including extant lizards, snakes, tuatara, crocodiles, birds, and turtles, were derived from ancestor whose temporal region was completely roofed by bone. Earliest diapsid reptiles such as *Petrolacosaurus* (Araeoscelidia) acquired two temporal openings (fenestrae) on either side of the skull (red vertical bar). Recent molecular phylogenies indicate that turtles (Testudines) were derived from diapsid ancestor, which would require secondary closure of temporal fenestrae (green vertical bar). The bone surrounding anteroventral border of the upper temporal fenestra and anterodorsal border of the lower temporal fenestra in diapsids: the postorbital was colored in pink. The bone surrounding posteroventral border of the upper temporal fenestra and posterodorsal border of the lower temporal fenestra in diapsids: the squamosal was colored in blue. The bone surrounding the anteroventral margin of the lower temporal fenestra in diapsids: the jugal was colored in yellow. Lizards do not have the lower temporal bar. Both upper and lower temporal bars are absent in snakes and birds. Other extinct diapsid lineages such as Ichthyosauria and Sauropterygia were not included in phylogeny for simplicity.

Morphology of the skull of reptiles, especially the temporal region is highly diverse (Figure 1). This morphological diversity observed in the temporal region is broadly categorized into three architectural patterns. The anapsid condition of the skull is observed in basal amniotes such as *Scutosaurus* and *Captorhinus*, and in turtles. In these animals, the temporal region of the skull is completely roofed by bones, without temporal openings (fenestrae). The synapsid condition of the skull is recognized in ancestral lineages of extant mammals and is characterized by the presence of a temporal fenestra at lower position of either side of the skull. The diapsid condition of the skull is seen in "non-turtle" extant reptiles and in their extinct relatives. In these animals, two temporal fenestrae exist on either side of the skull. In reptiles with fully diapsid skulls, the upper temporal fenestra is dorsally bordered by the parietal bone, anteriorly by the postorbital bone, and posteriorly by the squamosal bone. The lower temporal fenestra is dorsally surrounded by both the postorbital and squamosal bones, and ventrally by both the jugal and quadratojugal bones. These temporal fenestrae are thought to have evolved to allow space for accommodating the enlarged jaw-closing muscles that enable powerful biting, or to minimize stresses exerted by the contraction of jaw muscles on the skull, or to reduce the weight of the skull itself (Frazzetta, 1968, Carroll, 1982, Rieppel, 1993a, Benton, 2005). During the course of diapsid evolution skull morphology has been rearranged repeatedly, resulting in a variety of modified patterns (Rieppel, 1993a;Rieppel & Gronowski,, Rieppel & Gronowski, Rieppel & Gronowski,^1981^;Müller,, Müller, Müller,^2003^, Moazen et al., 2009). Among extant reptiles, fully diapsid skull is only seen in tuatara and crocodiles. Because the lower temporal bar that encloses the lower temporal fenestra ventrally is regarded to be lost once in the common ancestor of lepidosaurs (lizards, snakes, tuatara) and archosaurs (crocodiles and birds) (Rieppel, 1993a;Müller,, Müller, Müller,^2003^), these reptilian lineages possibly acquired the lower temporal bar secondarily (Müller, 2003, Moazen et al., 2009). Both snakes and birds have lost the upper temporal bar so that their temporal region is free from any bony frames (Pough et al., 2005).

In reptiles, phylogenetic position of turtles is highly controversial. Traditionally, turtles have been regarded as the only surviving clade of stem reptiles based on the pattern of their skull morphology: an anapsid skull whose temporal region is completely roofed with bones (Williston, 1917, Gregory, 1946, Romer, 1968, Gaffney, 1980;Reisz & Laurin,, Reisz & Laurin, Reisz & Laurin,^1991^, Lee, 1993;Laurin & Reisz,, Laurin & Reisz, Laurin & Reisz,^1995^, Lee, 1996,1997, Reisz, 1997, Lee, 2001). However, recent comprehensive analysis of morphological traits (Rieppel & deBraga, 1996;deBraga & Rieppel,, deBraga & Rieppel, deBraga & Rieppel,^1997^, Rieppel, 2000, Hill, 2005, Li et al., 2008, but see Lyson et al., 2010,2013 for opposed conclusion) and molecular phylogenetic studies (Hedges & Poling, 1999;Kumazawa & Nishida,, Kumazawa & Nishida, Kumazawa & Nishida,^1999^, Iwabe et al., 2005, Hugall et al., 2007, Shedlock et al., 2007, Shen et al., 2011, Tzika et al., 2011, Chiari et al., 2012, Crawford et al., 2012, Fong et al., 2012, Lyson et al., 2012, Wang et al., 2013) suggest that there is a close relationship of turtles to diapsid reptiles, implying that the temporal fenestrae were secondarily closed in turtles. In this study, we employ the hypothesis that turtles are descendent of diapsid reptiles.

Although skull morphology has been regarded as an important character in classification of reptiles and in understanding the ecological and physiological aspects of each reptilian species, the developmental mechanism underlying diversification of reptilian skull morphology is poorly understood (Rieppel, 1993a, Evans, 2008). As a consequence, a general genetic and developmental model of reptile skull diversity does not yet exist. In this paper, we test the hypothesis that changes of skeletal gene expression patterns cause diversification of reptilian skull morphology through comparative analyses of gene expression in the embryos of representative reptilian species and reveal a potential developmental basis underlying reptilian skull evolution. First, we describe the pattern of early phases of cranial morphogenesis in a crocodile species with both upper and lower temporal bars surrounding temporal fenestrae, using molecular markers specific for musculoskeletal tissue precursors. Then, we compare these data with cranial morphogenesis in a turtle species. We found a broader expression of the early osteogenic genes, *Runx2* and *Msx2* in the mesenchymal cells at the temporal region of turtle embryos, compared to that in crocodile embryos. Finally, to obtain a broader picture of reptilian skull morphogenesis, we examined expression patterns of *Runx2* and *Msx2* in cranial morphogenesis of a snake species without temporal bars on the skull and compared with the patterns in crocodile and turtle embryos. Our findings suggest that there is a possible correlation between the expression patterns of *Runx2* and *Msx2* and the architectural pattern seen in the temporal region of the reptilian skull.

## Results

In previous studies in which cranial osteogenesis of reptilian embryos was described, whole-mount clearing and staining with Alizarin red was used to detect mineralization of intramembranous bones that comprise the dermatocranium (Kamal et al., 1970;Haluska & Alberch,, Haluska & Alberch, Haluska & Alberch,^1983^, Rieppel, 1993b, Rieppel, 1993c, Kuratani, 1999;Rieppel & Zaher,, Rieppel & Zaher, Rieppel & Zaher,^2001^, Sheil, 2003, Sheil, 2005, Boughner et al., 2007;Vickaryous & Hall,, Vickaryous & Hall, Vickaryous & Hall,^2008^;Sánchez-Villagra et al.,, Sánchez-Villagra et al., Sánchez-Villagra et al.,^2009^, Werneburg et al., 2009). However, this method is unable to identify the distribution of the precursor cells of bones: osteoblasts, as reported by others (Kerney et al., 2010). To overcome this, we conducted section *in situ* hybridization analysis, which labels tissues located deep inside of the embryonic body and is effective for detecting tissue-specific domains of expression. We used a probe to *Runx2*, which is a molecular marker for osteogenic mesenchymal precursor cells (Ducy et al., 1997, Bobola et al., 2003, Abzhanov et al., 2007, Han et al., 2007, Kerney et al., 2010) and described its expression pattern in the temporal region of reptilian embryos where mineralization of bones has not been initiated. Furthermore, to describe distribution pattern of "non-osteoblast" cell lineages relative to that of osteoblasts in the cranial tissue of the embryos, we also examined expression of other tissue-specific markers: *MyoD* for skeletal muscle precursors (Hacker & Guthrie, 1998, Noden et al., 1999), *Sox9* for cartilage precursors (Wright et al., 1995, Bell et al., 1997), *Scleraxis* (*Scx*) and *Six2* for precursor of connective tissues, including ligaments and tendons (Oliver et al., 1995, Schweitzer, 2001, Dreyer et al., 2004;Edom-Vovard & Duprez,, Edom-Vovard & Duprez, Edom-Vovard & Duprez,^2004^, Schweitzer et al., 2010).

### Differential expression of early osteoblast marker, *Runx2*, in the head of crocodile and turtle embryos

**Figure 2:**
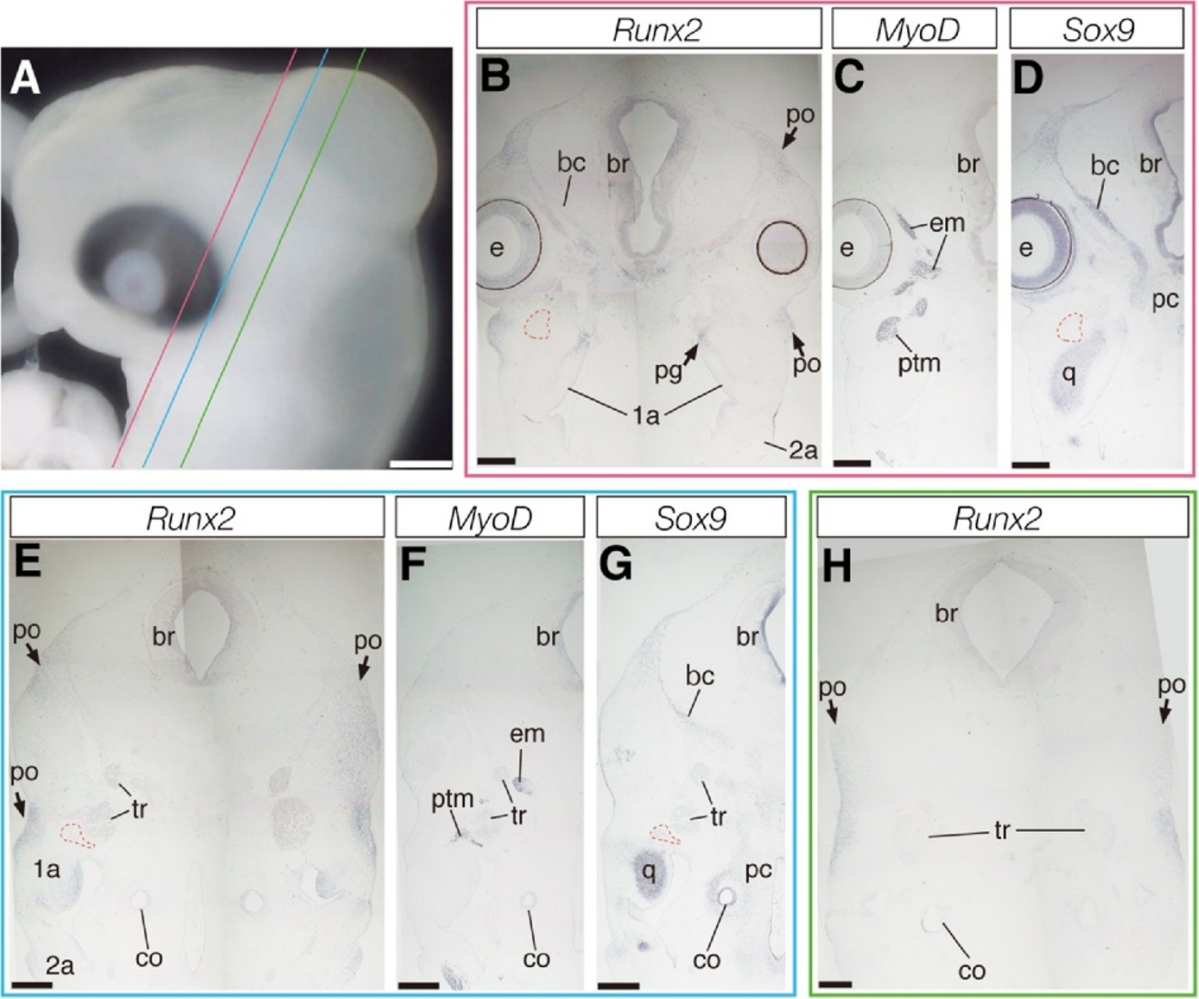
**Expression of musculoskeletal tissue marker genes in the head of crocodile embryos at stage 15.** **(A)** Lateral view of the head of a crocodile embryo at stage 15. **(B-D)** Frontal sections prepared around the plane indicated by the red line in **(A)**. **(E-G)** Frontal sections prepared around the plane indicated by the blue line in **(A)**. **(H)** Frontal sections prepared around the plane indicated by the green line in **(A)**. **(B, E, and H)** *Runx2*-positive mesenchymal cells are observed at the location where future dermatocranial elements are developed. **(C and F)** Expression of *MyoD* is detected at the cranial muscular tissues. **(D and G)** Cartilaginous tissues, including the braincase, quadrate, and Meckel's, are clearly labeled by *Sox9* probe. The red outlined domains in **(B, D, E, and G)** indicate the location of the pseudotemporal muscle (ptm) deduced from adjacent sections where muscular tissues are labeled by *MyoD* probe. Scale bar in **(A)** is 1 mm. Scale bars in **(B-H)** are 0.5 mm.

Osteogenic mesenchymal precursor cells that express *Runx2* are first detected in the temporal region of crocodile embryos at stage 14 (Additional file 1) and an almost identical pattern of *Runx2* expression was observed in a subsequent embryonic stage (stage 15; Figure 2A). These *Runx2*-positive cells were localized at the domain dorsal to the oral cavity where the ventral part of the braincase and future palatine and pterygoid bones develop, as well as in a limited domain dorsolateral to the orbit where the future dorsal projection of the postorbital bone forms (Figure 2B). We also detected a population of *Runx2*-positive cells at the domain ventrolateral to the orbit where future jugal bone and ventral projection of postorbital bone are formed. At this stage, the precursor of the jugal and ventral projection of the postorbital were dorsoventrally continued as a layer of cells but it was thin mediolaterally, especially at the middle part. In the posterior part of the head, we observe a population of *Runx2*-positive mesenchymal cells that later differentiate into the main body of the postorbital bone (Figures 2E and 2H). In these stages of crocodile embryogenesis, jaw muscle precursors that were derived from cranial mesoderm migrated to the first pharyngeal arch and expressed *MyoD* was clearly detected at the central domain of the jaw primordia (Figures 2C and 2F; Additional file 1). Expression of *Sox9* was detected at cartilage precursors that later differentiate into quadrate and Meckel's cartilages at the domain ventral to jaw muscle precursor, as well as in chondrocytes that form the future braincase (Figures 2D and 2G). Expression of *Scx* was detected in tendon precursor cells that are distributed within the primordia of the jaw muscles and in the connective tissue within the eye muscles (Additional file 1). Expression of *Six2* was somewhat broader than that of other markers, expressed in mesenchymal cells surrounding the eyes, cartilaginous precursors of the braincase, quadrate, and Meckel's, as well as in the mesenchyme at the interface between muscle precursors and the skeletal tissues to which the muscles attach and in a population of the mesenchyme that dorsally surrounds the brain (Additional file 1).

**Figure 3:**
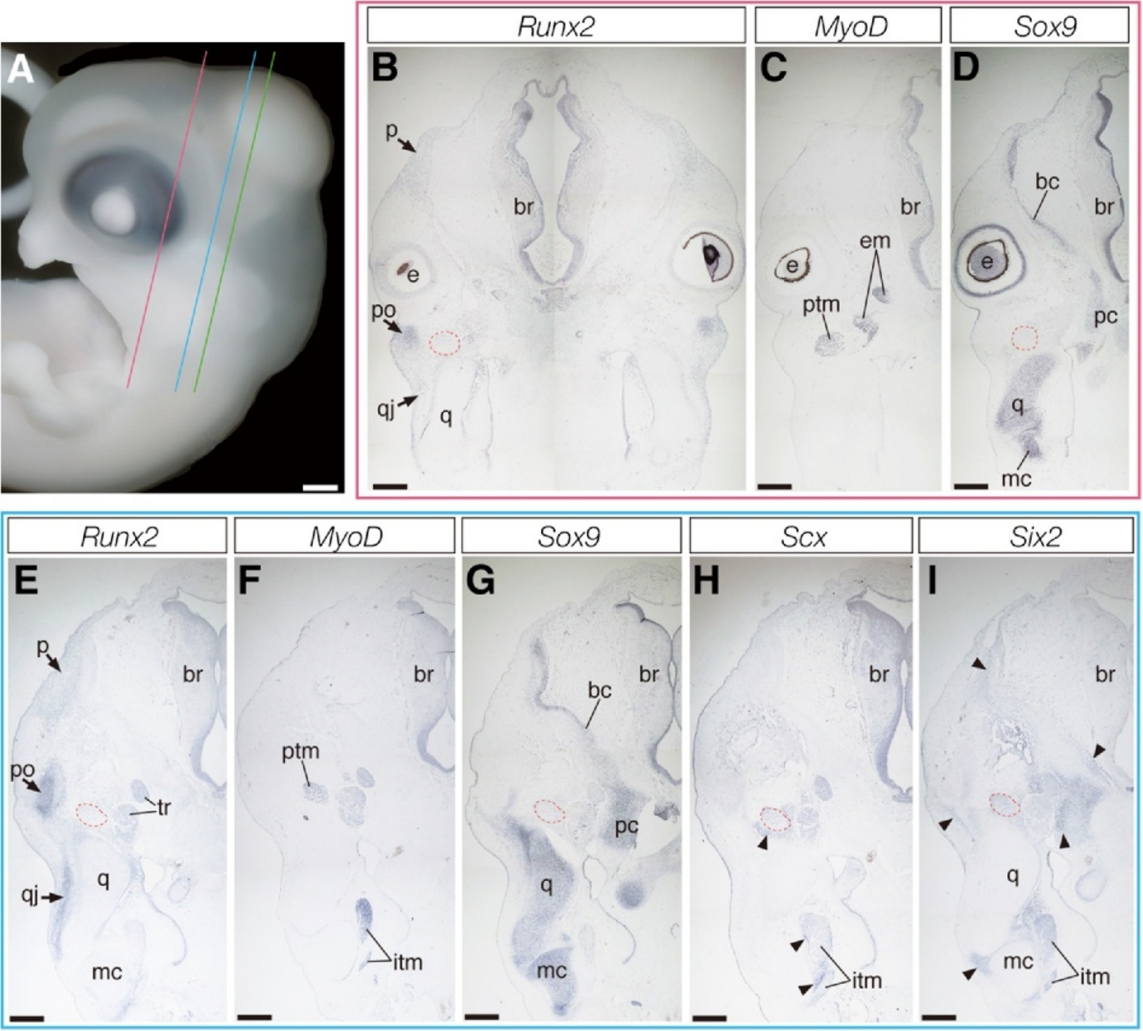
**Expression of musculoskeletal tissue marker genes in the head of crocodile embryos at stage 17.** **(A)** Lateral view of the head of a crocodile embryo at stage 17. **(B-D)** Frontal sections prepared around the plane indicated by the red line in **(A)**. **(E-I)** Frontal sections prepared around the plane indicated by the blue line in **(A)**. **(B and E)** Expression of *Runx2* is more concentrated to the precursors of dermatocranial elements, compared to the previous stages. **(C, D, F, and G)** Cranial muscular and cartilaginous tissues are clearly labeled by *MyoD* and *Sox9* probes, respectively. **(H and I)** Expression domains of *Scx* and *Six2* are indicated by arrowheads. The former is expressed in tendinous tissues accompanying cranial muscles and the latter is expressed mainly in connective tissue cells associated with cartilages of the jaw and the braincase. The red outlined domains in **(B, D, E, G, H, and I)** indicate the location of the pseudotemporal muscle deduced from adjacent sections where muscular tissues are labeled by *MyoD* probe. Green line in **(A)** indicates the plane where sections given in Figure 7D, E and F were prepared. Scale bar in **(A)** is 1 mm. Scale bars in **(B-I)** are 0.5 mm.

In crocodile embryos at stage 17 where none of the dermatocranial elements were positive for Alizarin red in previous studies (Rieppel, 1993b;Vickaryous & Hall,, Vickaryous & Hall, Vickaryous & Hall,^2008^) (Figure 3A), we could detect *Runx2* expression in the cell populations that were localized to the area where the future dermatocranium differentiates (Figure 3B). Although the domain where *Runx2*-positive cells were populated was almost identical to that in previous stages, the boundary of each precursor of the dermatocranial elements became clearer. Although differentiation of the parietal bone is delayed compared to other dermatocranial elements as described previously (Rieppel 1993b;Vickaryous & Hall,, Vickaryous & Hall, Vickaryous & Hall,^2008^), a pair of precursors of the parietal were recognized as *Runx2*-positive cell aggregation at the domain dorsolateral to the cartilaginous braincase (Figures 3B and 3E). We could detect *Runx2*-negative cell populations between the nascent parietal precursor and the morphologically more developed postorbital precursor and also between precursors of the postorbital and quadratojugal located lateral to *Sox9*-positive quadrate cartilage (Figure 3E). In this stage, *MyoD* was expressed in differentiated jaw and eye muscles (Figures 3C and 3F) and *Sox9* was expressed in differentiated chondrocranium and splanchnocranium components, including the braincase, quadrate, and Meckel's (Figures 3D and 3G). Expression of *Scx* was detected in tendinous tissues accompanying *MyoD*-positive muscles as in previous stages (Figure 3H). Expression of *Six2* was detected in the mesenchyme localized around the jaw articulation between quadrate and Meckel's, as well as in adjacent mesenchyme of the braincase, postorbital bone, and jaw muscles (Figure 3I).

**Figure 4:**
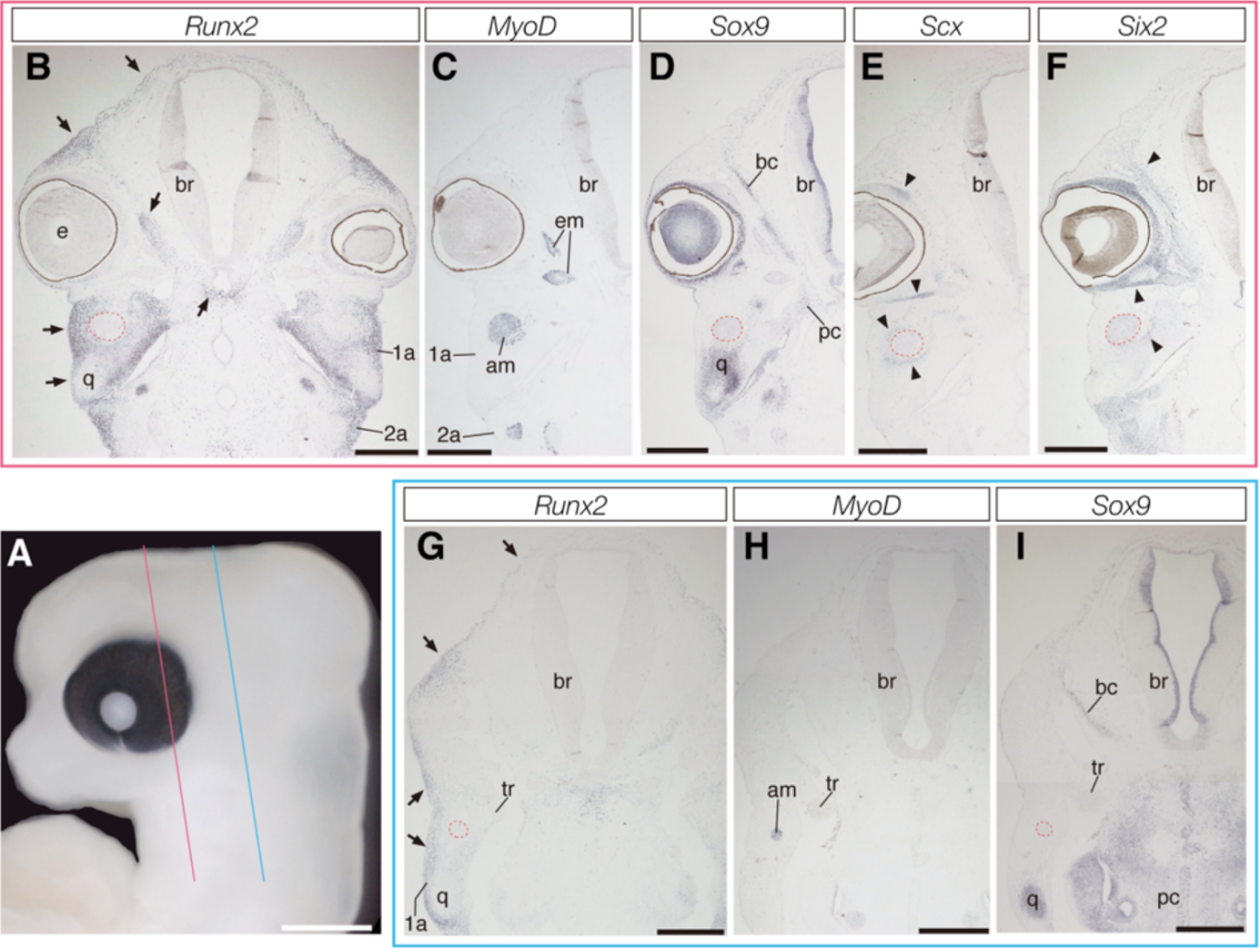
**Expression of musculoskeletal tissue marker genes in the head of turtle embryos at stage 15.** **(A)** Lateral view of the head of a turtle embryo at stage 15. **(B-F)** Frontal sections prepared around the plane indicated by the red line in **(A)**. **(G-I)** Frontal sections prepared around the plane indicated by the blue line in **(A)**. **(B, G)** *Runx2*-positive mesenchymal cells (arrows) are broadly distributed at lateral portion of the head, from the top of the head to the ventral margin of the first pharyngeal arch. **(C and H)** Cranial muscular tissues are clearly labeled by *MyoD* probe. **(D and I)** Cartilaginous tissues, including the braincase and quadrate, are clearly labeled by *Sox9* probe. **(E)** *Scx* is expressed in tendon primordia accompanying cranial muscles (arrowheads). **(F)** *Six2* is expressed mainly in connective tissue cells associated with the braincase cartilage and jaw adductor muscle (arrowheads). Note that the anlage of jaw adductor muscle (red outlined domain) is covered by a thick layer of *Runx2*-positive mesenchyme laterally. Scale bar in **(A)** is 1 mm. Scale bars in **(B-I)** are 0.5 mm.

Next, we examined cranial morphogenesis of turtles that have an anapsid skull, using the same method to identify the distribution pattern of precursors of each tissue that constitutes the cranial musculoskeletal system. In turtle embryos at stage 14 (Additional file 2) that correspond to crocodile embryos at stage 14 in external morphology, and in turtle embryos at stage 15 (Figure 4A) that are comparable to crocodile embryos at stage 15, we observed almost identical patterns of expression for each gene. We detected *MyoD* expression specifically in the primordia of jaw adductor and eye muscles (Figures 4C and 4H; Additional file 2) and *Sox9* expression in precursor cells of the braincase, quadrate, and Meckel's cartilages (Figures 4D and 4I; Additional file 2) as in stage-matched crocodile embryos. Expression of *Scx* was first detected in a layer of mesenchymal cells that was located at the periphery of the jaw adductor and eye muscle precursors in stage 15 turtle embryos (Figure 4E). Expression of *Six2* was observed in the mesenchyme surrounding the eye and adjacent mesenchyme of the braincase and jaw cartilages, as well as in some mesenchymal cells within jaw muscle precursors (Figure 4F; see Additional file 2), as in stage-matched crocodile embryos. Interestingly, we observed expression of the early osteoblast marker, *Runx2*, in a broader domain at the temporal region of the head of turtle embryos, compared to that in stage-matched crocodile embryos. In stage14 turtle embryos, *Runx2* expression was detected in a population of cells medial to the precursor of the jaw adductor muscles and the mesenchyme localized at the domain dorsolateral and ventrolateral to the orbit (Additional file 2). The domain of *Runx2* expression became further expanded in the head of turtle embryos at stage 15. A thick layer of the mesenchymal cells that express *Runx2* completely covered the brain and the precursor of jaw adductor muscle laterally (Figures 4B and 4G).

**Figure 5:**
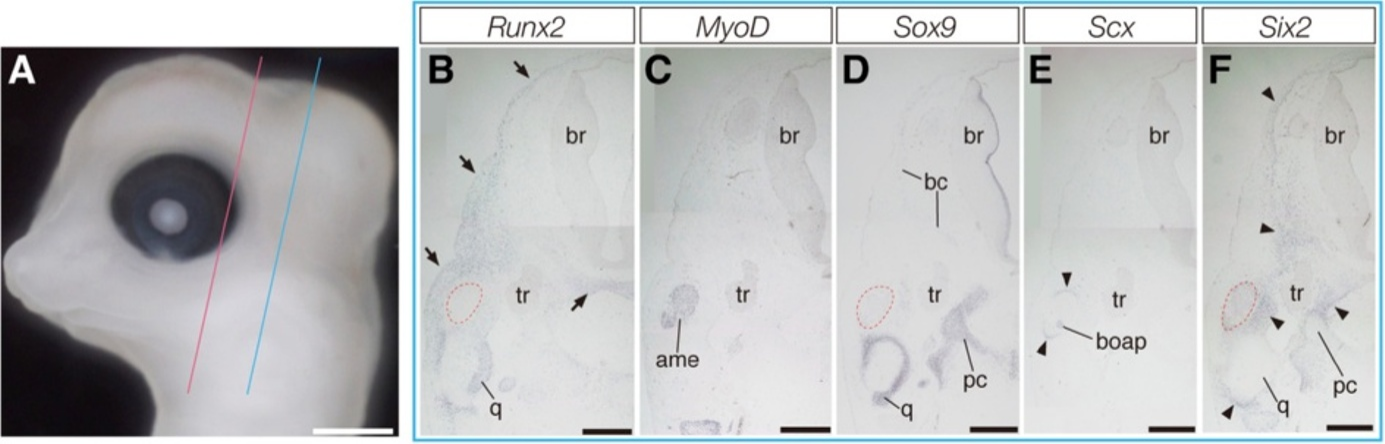
**Expression of musculoskeletal tissue marker genes in the head of turtle embryos at stage 17.** **(A)** Lateral view of the head of a turtle embryo at stage 17. **(B-F)** Frontal sections prepared around the plane indicated by the blue line in **(A)**. **(B)** *Runx2*-positive mesenchymal cells (arrows) are broadly distributed at the lateral portion of the head, from the top of the head to the ventral margin of the first pharyngeal arch derivative. **(C)** Cranial muscular tissues are clearly labeled by *MyoD* probe. **(D)** Cartilaginous tissues, including the braincase and quadrate, are clearly labeled by *Sox9* probe. **(E)** *Scx* is expressed in tendinous tissues at the periphery of external adductor muscle (ame) (arrowheads) and in the bodenaponeurosis (boap. central tendon of jaw adductor muscle). **(F)** *Six2* is expressed mainly in connective tissue cells associated with cartilages of the braincase and quadrate, as well as in connective tissue cells within jaw muscles (arrowheads). Note that the external adductor muscle (red outlined domain) is covered by a thick layer of *Runx2*-positive mesenchyme laterally. Scale bar in **(A)** is 1 mm. Scale bars in **(B-F)** are 0.5 mm.

In turtle embryos at stage 17 (Figure 5A) that correspond to crocodile embryos at stage 17 in overall morphology, we observed *MyoD* expression in differentiating cranial muscles, including external adductor muscles (Figure 5C) and *Sox9* expression in the cartilaginous tissues that constitute the braincase, quadrate, and Meckel's (Figure 5D). *Scx* was specifically expressed in tendinous tissues at the periphery of jaw adductor muscles, as well as in the precursor of the bodenaponeurosis (central tendon of external adductor) just appeared within the jaw adductor muscular tissue (Figure 5E). The expression domain of *Six2* was broader in the temporal region of the head compared to that of *Scx*, diffusively expressed in the mesenchymal cells surrounding jaw adductor muscles, braincase, and jaw cartilages (Figure 5F). A thick layer of *Runx2*-positive mesenchymal cells that surrounds the braincase and jaw adductor muscle laterally was observed (Figure 5B). *Runx2*-expressing mesenchyme was also distributed around the quadrate cartilage and the ventral part of the braincase.

### Expression of potential upstream osteogenic regulatory genes in the head of crocodile and turtle embryos

Through comparative analysis of expression patterns of tissue-specific marker genes, we noticed a difference in the spatial pattern of expression of the early osteoblast marker, *Runx2* in the head of crocodile and turtle embryos. To reveal potential mechanisms that account for such differential distribution of osteogenic mesenchymal precursor cells between two reptilian lineages with or without temporal fenestrae, we next examined expression patterns of some candidate genes that are known to regulate cranial osteogenesis. In the present study, we focused on *Bmp4*, *Msx1*, and *Msx2*. Bmp4 is a signaling molecule and plays a key role in the Bmp signaling pathway. Because exogenous Bmp4 increases tissue volume in calvarial bone tissue culture, this protein is considered to be involved in calvarial bone growth (Kim et al., 1998, Rice et al., 2003). Both Msx1 and Msx2 are members of the muscle segment homeobox (msh) gene family of transcription factors and both loss-of- and gain-of-function analyses of these genes suggest their essential roles in vertebrate cranial osteogenesis (Satokata & Maas, 1994, Satokata et al., 2000).

**Figure 6:**
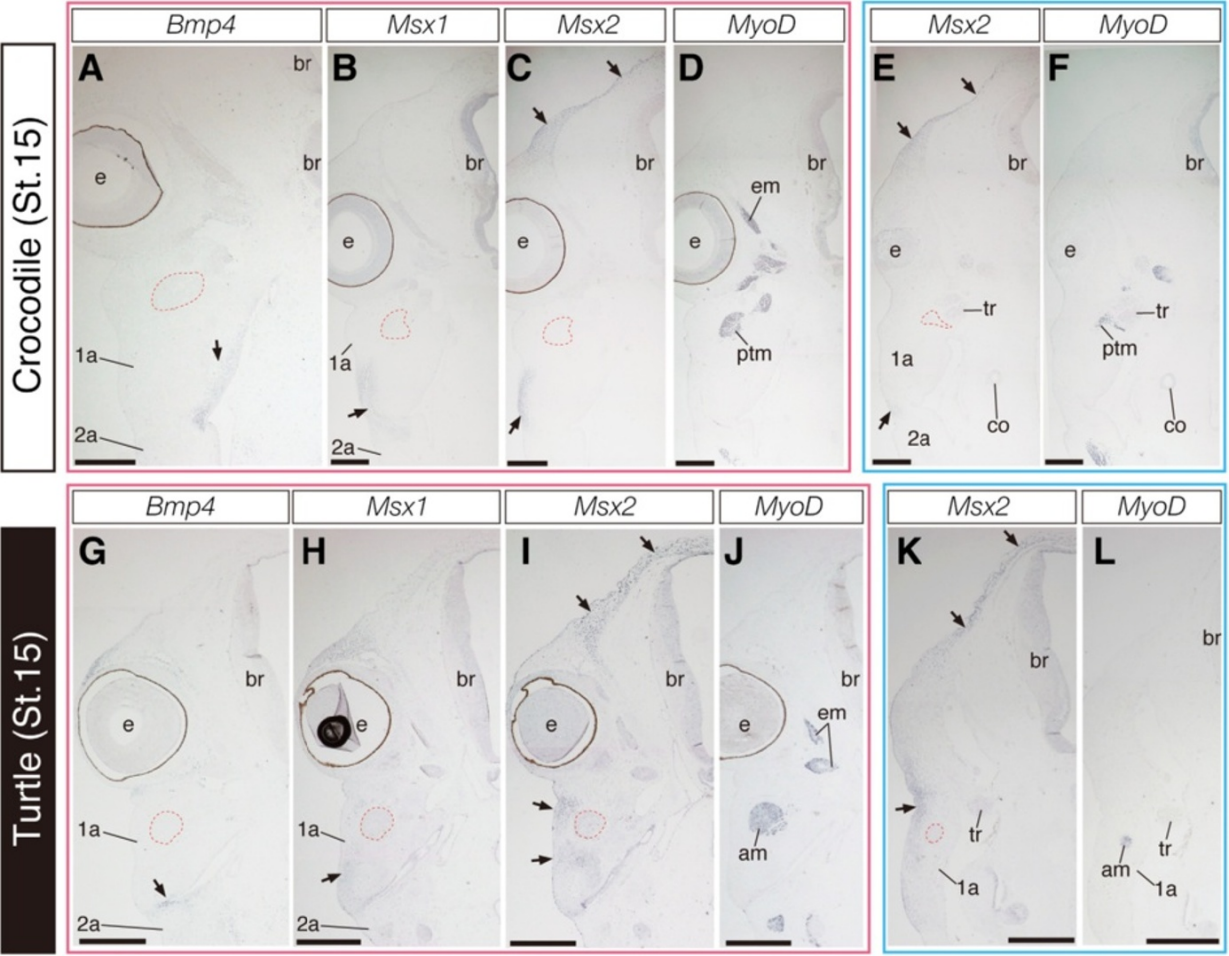
**Expression of** ***Bmp4*** **,** ***Msx1*** **, and** ***Msx2*** **in crocodile and turtle embryos at stage 15.** **(A-D)** Frontal sections prepared around the plane indicated by the red line in Figure 2A. **(E, F)** Frontal sections prepared around the plane indicated by the blue line in Figure 2A. **(G-J)** Frontal sections prepared around the plane indicated by the red line in Figure 4A. **(K, L)** Frontal sections prepared around the plane indicated by the blue line in Figure 4A. **(A and G)** In both crocodile and turtle embryos, expression of *Bmp4* is detected at the mesenchyme distributed in medial part of the jaw primordia (arrows). **(B and H)** Expression of *Msx1* is detected at the mesenchyme that occupies the domain close to jaw articulation and lateral to quadrate and Meckel's cartilages (arrows). **(C and E)** In crocodile embryos, *Msx2* is expressed in a thin layer of mesenchymal cells surrounding dorsal aspect of the brain and in a population of the mesenchyme that occupies the domain between ventrolateral part of quadrate cartilage and surface epidermis (arrows). **(I and K)** In turtle embryos, *Msx2* is expressed in mesenchymal cells that populate lateral aspect of the head (arrows). In contrast to the condition in crocodile embryos, the ventral edge of *Msx2*-expressing mesenchymal layer is terminated ventral to the eye in turtle embryos and these cells cover *MyoD*-expressing jaw adductor muscle precursor **(J and L)** laterally. The red outlined domains in **(A-C, and E)** indicate the location of the anlagen of the pseudotemporal muscle deduced from adjacent sections where muscular tissues are labeled by *MyoD* probe. The red outlined domains in **(G-I, and K)** indicate the location of the anlagen of jaw adductor muscle. Scale bars are 0.5 mm.

**Figure 7:**
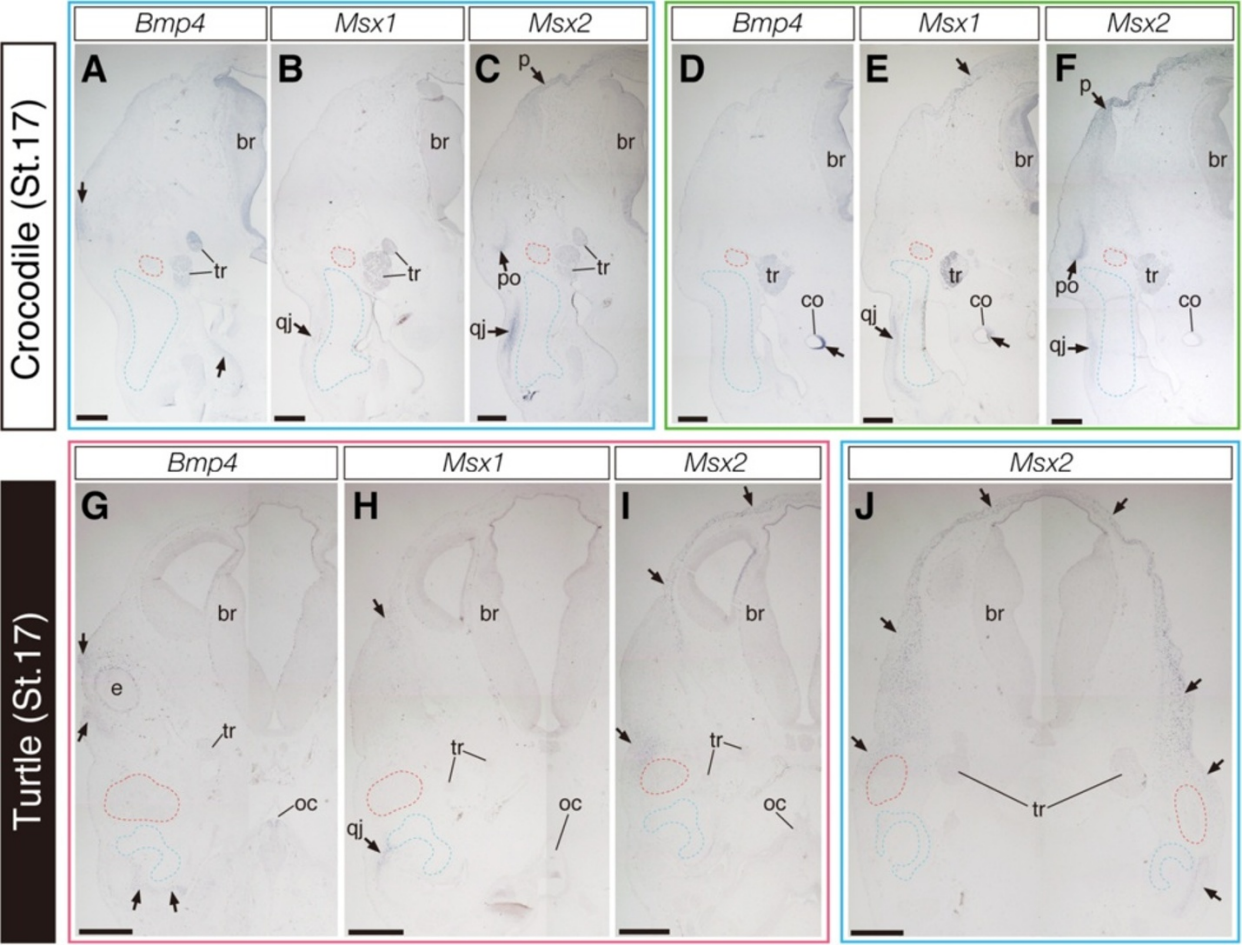
**Expression of** ***Bmp4*** **,** ***Msx1*** **, and** ***Msx2*** **in crocodile and turtle embryos at stage 17.** **(A-C)** Frontal sections prepared around the plane indicated by the blue line in Figure 3A. **(D-F)** Sections prepared around the plane indicated as the green line in Figure 3A. **(G-I)** Sections prepared around the plane indicated as the red line in Figure 5A. **(J)** Section prepared around the plane indicated as the blue line in Figure 5A. **(A, D, and G)** In both crocodile and turtle embryos, expression of *Bmp4* is detected in the epithelium of the cochlear canal, the mesenchyme surrounding the eye, and the mesenchyme distributed in medial part of the jaw (arrows). **(B, E, and H)** Expression of *Msx1* is detected at the mesenchymal cells that later differentiates into quadratojugal bone (qj) and in a thin layer of mesenchymal cells that covers brain dorsally (arrows at the top of E and H), as well as in the epithelium of the cochlear canal. **(C and F)** In crocodile embryos, expression of *Msx2* is detected at a population of mesenchymal cells in close proximity of postorbital and quadratojugal bones, as well as in a layer of the mesenchyme surrounding the brain dorsally where future a pair of parietal bones are developed. **(I and J)** In turtle embryos, *Msx2* was expressed in a thick layer of mesenchymal cells that populate lateral aspect of the head (arrows). The *Msx2*-positive mesenchymal cells cover the external adductor muscle precursors (red outlined domains in **G-** **J**) laterally. The red outlined domains in **(A-F)** indicate the location of the pseudotemporal muscle deduced from adjacent sections where muscular tissues are labeled by *MyoD* probe. The blue outlined domains in **(A-J)** indicate the location of quadrate cartilage deduced from adjacent sections where cartilaginous tissues are labeled by *Sox9* probe. Scale bars are 0.5 mm.

In the present analysis, we found that *Bmp4* and *Msx1* showed almost identical expression patterns through cranial osteogenesis in crocodile and turtle embryos. In crocodile embryos we examined (through stage 14 to stage 17), *Bmp4* was strongly expressed in the epithelium of cochlear canal, the mesenchyme surrounding the eye, the mesenchyme distributed in the medial part of jaw primordia, the precursors of the palatine bones, and a population of mesenchymal cells that covered the brain dorsally (Figure 6A; Figures 7A and 7D). In turtle embryos we examined (through stage 14 to stage 17), *Bmp4* was expressed in a spatially limited domain: the epitthelium of cochlear canal, the mesenchyme dorsolateral and ventrolateral to the eye and a limited population of the mesenchyme in close proximity of the jaw articulation (Figure 6G; Figure 7G). We observed *Msx1* expression in the epithelium of the cochlear canal, the mesenchyme that occupies the domain close to the jaw articulation and lateral to the quadrate and Meckel's cartilages, and a thin layer of mesenchymal cells that covers the brain dorsally in crocodile embryos examined (Figure 6B; Figures 7B and 7E). In turtle embryos, *Msx1* was expressed in the epithelium of the cochlear canal, the mesenchyme distributed around the jaw articulation and lateral to quadrate and Meckel's cartilages, as well as in the mesenchyme that populates the domain dorsal to the eye (Figure 6H; Figure 7H).

In contrast to *Bmp4* and *Msx1*, we detected differential expression patterns of *Msx2* in the head of crocodile and turtle embryos. In crocodile embryos at stage 14 and 15, *Msx2* was expressed in a thin layer of mesenchymal cells surrounding the dorsal aspect of the brain (Figures 6C and 6E). In the posterior part of the head, the ventral edge of this *Msx2*-expressing cell population is located dorsal to the eye. In these crocodilian embryos, *Msx2* expression was also observed in a population of the mesenchyme that occupied the domain between the ventrolateral part of quadrate cartilage and surface epidermis (Figures 6C and 6E). These mesenchymal cells expressed *Msx1* as well (Figure 6B) and appeared to differentiate into the quadratojugal bone later. In crocodile embryos at stage 17, specific expression of *Msx2* was detected at a population of mesenchymal cells in close proximity of *Runx2*-expressing precursors of postorbital and quadratojugal bones, as well as in a thin layer of the mesenchyme surrounding the brain dorsally where future parietal bones were developed (Figures 7C and 7F). We observed that the space adjacent to *Msx2*-positive precursors of these dermatocranial elements was filled with *Msx2*-negative mesenchymal cells. Interestingly, we observed broader expression of *Msx2* in turtle embryos, compared to that in stage-matched crocodile embryos. In turtle embryos examined, *Msx2* was expressed in mesenchymal cells that populate lateral aspect of the head of embryos (Figures 6I and 6K; Figures 7I and 7J). The ventral edge of the *Msx2*-expressing mesenchymal layer was terminated ventral to the eye and these cells covered *MyoD*-expressing external adductor muscle laterally. Showing its dorsoventrally broadened expression pattern, the domain of *Msx2* expression largely overlapped with that of *Runx2* in turtle embryos (Figures 4B and 4G; Figure 5B).

### Expression of *Runx2* and *Msx2* in the head of snake embryos

**Figure 8:**
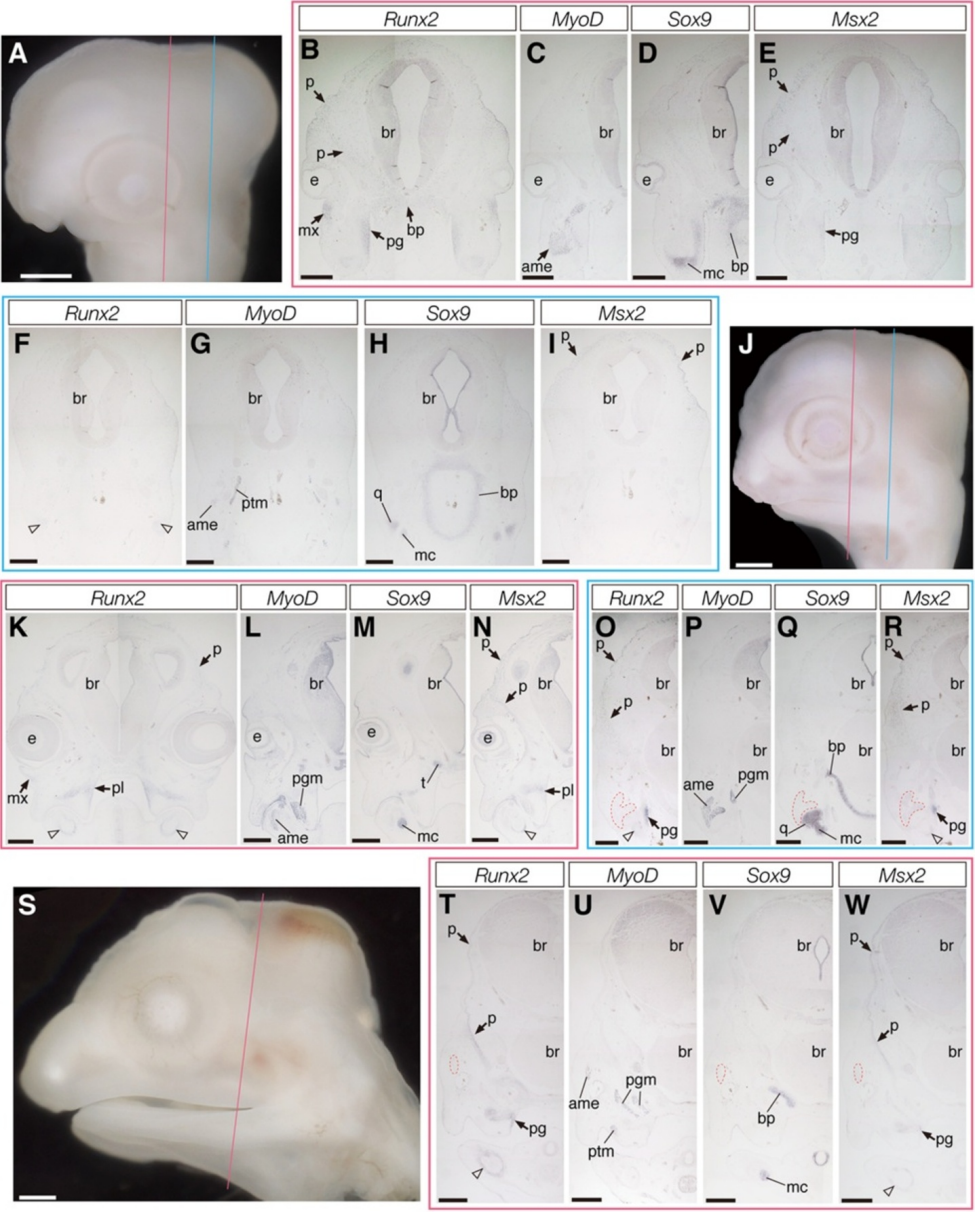
**Expression of the genes that regulate the development of cranial musculoskeletal tissues in snake embryos.** **(A)** Lateral view of the head of a snake embryo at stage 26. **(B-E)** Frontal sections prepared around the plane indicated by the red line in **(A)**. **(F-I)** Frontal sections around the blue line in **(A)**. **(J)** The head of a snake embryo at stage 29. **(K-N)** Frontal sections around the red line in **(J)**. **(O-R)** Frontal sections around the blue line in **(J)**. **(S)** The head of a snake embryo at stage 31. **(T-W)** Frontal sections around the red line in **(S)**. **(B, F, K, O, and T)** Expression of *Runx2*. **(C, G, L, P, and U)** Expression of *MyoD*. **(D, H, M, Q, and V)** Expression of *Sox9*. **(E, I, N, R, and W)** Expression of *Msx2*. In snake embryos, expression of *Runx2* and *Msx2* are detected in the precursor cells of dermatocranial elements (arrows), as in crocodile and turtle embryos. However, these mesenchymal cells are not seen in the temporal region lateral to the jaw adductor muscles. Instead, these cells are distributed in the vicinity of the brain, laterally covering it, and differentiate into the parietal bone in older embryos. Open arrowheads in **(F, K, N, O, R, T, and W)** indicate the mesenchyme around *Sox9*-positive jaw cartilages, where the expression of *Runx2* and *Msx2* is detected. The red outlined domains in **(O, Q, R, T, V, and W)** indicate the location of the external adductor muscle deduced from adjacent sections where muscular tissues are labeled by *MyoD* probe. Scale bars in **(A)**, **(J)**, and **(S)** are 1 mm. Scale bars in other pictures are 0.5 mm.

To verify a correlation between the expression patterns of *Runx2* and *Msx2* and reptilian skull morphology, we finally examined expression patterns of these genes, as well as of marker genes for muscular and cartilaginous tissues, in cranial morphogenesis of a snake species where their temporal fenestrae are not encircled by the temporal bars. In snake embryos at stage 26 (Figure 8A) that morphologically correspond to crocodile and turtle embryos at stage 14 or 15, *MyoD* was expressed in the primordia of the first arch muscles (Figures 8C and 8G). *Sox9* was strongly expressed in the precursors of quadrate and Meckel's cartilages and the base of the braincase, as well as in a layer of mesenchyme surrounding the brain laterally (Figures 8D and 8H). In these snake embryos, early osteoblast marker, *Runx2* was expressed in the mesenchyme that occupied the space medial to the *Sox9*-positive quadrate precursor and in a limited population of mesenchymal cells ventral to the orbit (Figure 8B). We also detected *Runx2* expression in a layer of mesenchymal cells that surround the brain laterally. In the posterior temporal region, *Runx2* was only faintly expressed in the adjacent mesenchyme of the *Sox9*-positive quadrate precursor (Figure 8F). Expression of *Msx2* was detected in the mesenchyme medial to the precursor of the quadrate (Figure 8E). Its expression domain was spatially overlapped with the domain where *Runx2*-positive cells were distributed, but the former was narrower. We also detected *Msx2* expression in a mesenchymal layer that surrounded the brain dorsally (Figures 8E and 8I). In snake embryos at stage 29 (Figure 8J), which morphologically correspond to crocodile and turtle embryos at stage 17, well-differentiated cranial muscular and cartilaginous tissues were specifically labeled by expression of *MyoD* (Figures 8L and 8P) and *Sox9* (Figures 8M and 8Q), respectively. We observed spatially overlapped expression of *Runx2* and *Msx2* in these embryos: both genes were expressed in the precursors of palatine and pterygoid bones medial to *Sox9*-positive jaw cartilages and in mesenchymal cells accompanying jaw cartilages, as well as in a layer of loose mesenchyme that later forms a precursor of parietal bones that cover the dorsal part of the brain (Figures 8K, 8N, 8O, and 8R). However, only *Runx2* was expressed in the mesenchyme that populated the domain ventral to the orbit, which possibly corresponds to precursors of the maxilla bones of the upper jaw. No *Runx2* and/or *Msx2* expressing osteogenic mesenchymal precursor cells populated the domain lateral to jaw adductor muscles. In more developed snake embryos (at stage 31; Figure 8S), both *Runx2* and *Msx2* were specifically expressed in the precursors of the dermatocranial elements, including the parietal that encases the brain dorsally (Figures 8T and 8W). As in previous stages, expression of the former was more expanded. The lateral aspect of the external adductor muscles was never covered by the skeletal tissues that express *Runx2* and/or *Msx2*. The results on expression domains of each gene analyzed in crocodile, turtle, and snake embryos are summarized in Table 1.

**Table 1:** **Expression domains of the genes in the head of crocodile, turtle, and snake embryos**

Genes	Crocodile	Turtle	Snake
*Runx2*	**St.14/15**: The domain dorsal to the oral cavity where the ventral part of the braincase and future palatine and pterygoid bones develop; a domain dorsolateral to the orbit where the future dorsal projection of the postorbital bone forms; the domain ventrolateral to the orbit where future jugal and postorbital bones form; the mesenchyme that later differentiates into the main body of the postorbital bone.	**St.14/15**: A population of cells medial to the precursor of the jaw adductor muscles; the mesenchyme localized at the domain dorsolateral and ventrolateral to the orbit; a thick layer of the mesenchymal cells that completely covers the brain and the precursor of jaw adductor muscle laterally.	**St.26**: The mesenchyme occupying the space medial to the quadrate cartilage precursor; the mesenchyme ventral to the orbit; a layer of mesenchymal cells surrounding the brain laterally.
	**St.17**: The cell populations localized to the area where the future dermatocranium differentiates (palatine, parietal, postorbital, pterygoid, quadratojugal bones).	**St.17**: A thick layer of mesenchymal cells surrounding the braincase and jaw adductor muscle laterally; the mesenchyme associated with the quadrate cartilage and the ventral part of the braincase.	**St.29**: The precursors of palatine and pterygoid bones; the mesenchyme accompanying jaw cartilages; a layer of loose mesenchyme that later forms a precursor of parietal bones; the precursors of the maxilla bones.
			**St.31**: The precursors of the dermatocranial elements, including the parietal.
*MyoD*	**St.14/15**: Precursor cells of each jaw muscle in the first pharyngeal arch; eye muscle precursors.	**St.14/15**: The primordia of jaw and eye muscles.	**St.26**: The primordia of jaw and eye muscles.
	**St.17**: Differentiated jaw and eye muscles.	**St.17:** Differentiated jaw and eye muscles.	**St.29-31**: Differentiated jaw and eye muscles.
*Sox9*	**St.14/15**: Cartilage precursors that later differentiate into the quadrate, Meckel's cartilage, and the braincase.	**St.14/15**: Precursor cells of the braincase, quadrate, and Meckel's cartilages.	**St.26**: The precursors of quadrate and Meckel's cartilages and the braincase; a layer of mesenchyme surrounding the brain laterally.
	**St.17**: Differentiated chondrocranium and splanchnocranium components (the braincase, quadrate, and Meckel's).	**St.17:** Differentiated chondrocranium and splanchnocranium components (the braincase, quadrate, and Meckel's).	**St.29-31**: Differentiated chondrocranium and splanchnocranium components (the braincase, quadrate, and Meckel's).
*Scx*	**St.14/15**: Tendon precursor cells within jaw muscle primordia; connective tissue within eye muscles.	**St.14/15**: A layer of mesenchymal cells located at the periphery of the jaw adductor and eye muscle precursors.	-
	**St.17**: Tendinous tissues accompanying jaw muscles; connective tissue associated with eye muscles.	**St.17:** Tendinous tissues at the periphery of jaw adductor muscles; the precursor of the bodenaponeurosis (central tendon of external adductor) within the jaw adductor muscular tissue.	
*Six2*	**St.14/15**: The mesenchyme surrounding the eyes and cartilaginous precursors of the braincase, quadrate, and Meckel's; the mesenchyme between jaw muscle precursors and the skeletal tissues to which the muscles attach; the mesenchyme that dorsally surrounds the brain.	**St.14/15**: The mesenchyme surrounding the eye; the mesenchyme associated with the braincase and jaw cartilages; the mesenchyme within the jaw muscle precursors.	-
	**St.17**: The mesenchyme localized around the jaw articulation between quadrate and Meckel's; the mesenchyme associated with the braincase, postorbital bone, and jaw muscles.	**St.17:** The mesenchyme surrounding jaw adductor muscles, braincase, and jaw cartilages.	
*Bmp4*	**St.14-17**: The epithelium of cochlear canal; the mesenchyme surrounding the eye; the mesenchyme distributed in the medial part of jaw primordia; the precursors of the palatine bones; a population of mesenchymal cells covering the brain dorsally.	**St.14-17**: The epithelium if cochlear canal; The mesenchyme dorsolateral and ventrolateral to the eye; a limited population of the mesenchyme in close proximity of the jaw articulation.	-
*Msx1*	**St.14-17**: The epithelium of the cochlear canal; the mesenchyme adjacent to the jaw articulation; the mesenchyme lateral to the quadrate and Meckel's cartilages; a thin layer of mesenchymal cells covering the brain dorsally.	**St.14-17**: The epithelium of the cochlear canal; the mesenchyme adjacent to the jaw articulation; the mesenchyme lateral to quadrate and Meckel's cartilages; the mesenchyme that populates the domain dorsal to the eye.	-
*Msx2*	**St.14/15**: A thin layer of mesenchymal cells surrounding the dorsal aspect of the brain; the mesenchyme located dorsal to the eye; the mesenchyme occupying the domain between the ventrolateral part of quadrate cartilage and surface epidermis.	**St.14-17**: Mesenchymal cells populating lateral aspect of the head (lateral to external adductor muscle).	**St.26**: The mesenchyme medial to the quadrate precursor; a mesenchymal layer surrounding the brain dorsally.
	**St.17**: A population of mesenchymal cells in close proximity of postorbital and quadratojugal bone precursors; a thin layer of the mesenchyme surrounding the brain dorsally where future parietal bones form.		**St.29**: The precursors of palatine and pterygoid bones; the mesenchyme accompanying jaw cartilages; a layer of loose mesenchyme that later forms a precursor of parietal bones.
			**St.31**: The precursors of the dermatocranial elements, including the parietal.

## Discussion

### Potential developmental basis of anapsid skull in turtles

Skull morphology, especially the osteological configuration of the temporal region, has historically been treated as the most important character in the classification of major lineages of reptiles. Based on their anapsid skull, turtles have been regarded as a sole descendent of stem reptiles (Williston, 1917, Gregory, 1946, Romer, 1968, Gaffney, 1980;Reisz & Laurin,, Reisz & Laurin, Reisz & Laurin,^1991^, Lee, 1993;Laurin & Reisz, 1995, Lee, 1996,1997, Reisz, 1997, Lee, 2001) despite the contrary argument that turtles were derived from an ancestor with a diapsid skull (Lakjer, 1926, Goodrich, 1930). Recent phylogenetic studies where the interrelationships of both extant and extinct reptiles were surveyed through comprehensive analysis of multiple osteological traits concluded that turtles were closely related to lepidosaurian diapsids (Rieppel & deBraga, 1996;deBraga & Rieppel , deBraga & Rieppel deBraga & Rieppel 1997Rieppel 2000, Hill, 2005, Li et al., 2008). Furthermore, results of molecular phylogenetic studies have strongly suggested diapsid affinity of turtles (Hedges & Poling, 1999;Kumazawa & Nishida,, Kumazawa & Nishida, Kumazawa & Nishida,^1999^, Iwabe et al., 2005, Hugall et al., 2007, Shedlock et al., 2007Shen et al. 2011, Tzika et al., 2011, Chiari et al., 2012, Crawford et al., 2012, Fong et al., 2012, Lyson et al., 2012, Wang et al., 2013). If turtles were derived from a diapsid ancestor, then the anapsid skull of turtles evolved independently from that of ancestral lineages of reptiles by secondary closure of the temporal fenestrae. However, although the phylogenetic position of turtles within amniotes still remains inconclusive (Lyson et al., 2010,2013, Kuratani et al., 2011), there has been no study in which the process of development of their anapsid skull is described with molecular markers for labeling precursor cells of the dermatocranium. In the present study, we examined early cranial morphogenesis of representative reptilian species through comparative analysis of gene expression patterns and found unique expression patterns of *Runx2* and *Msx2* in turtle embryos that are not observed in crocodile and snake embryos.

**Figure 9:**
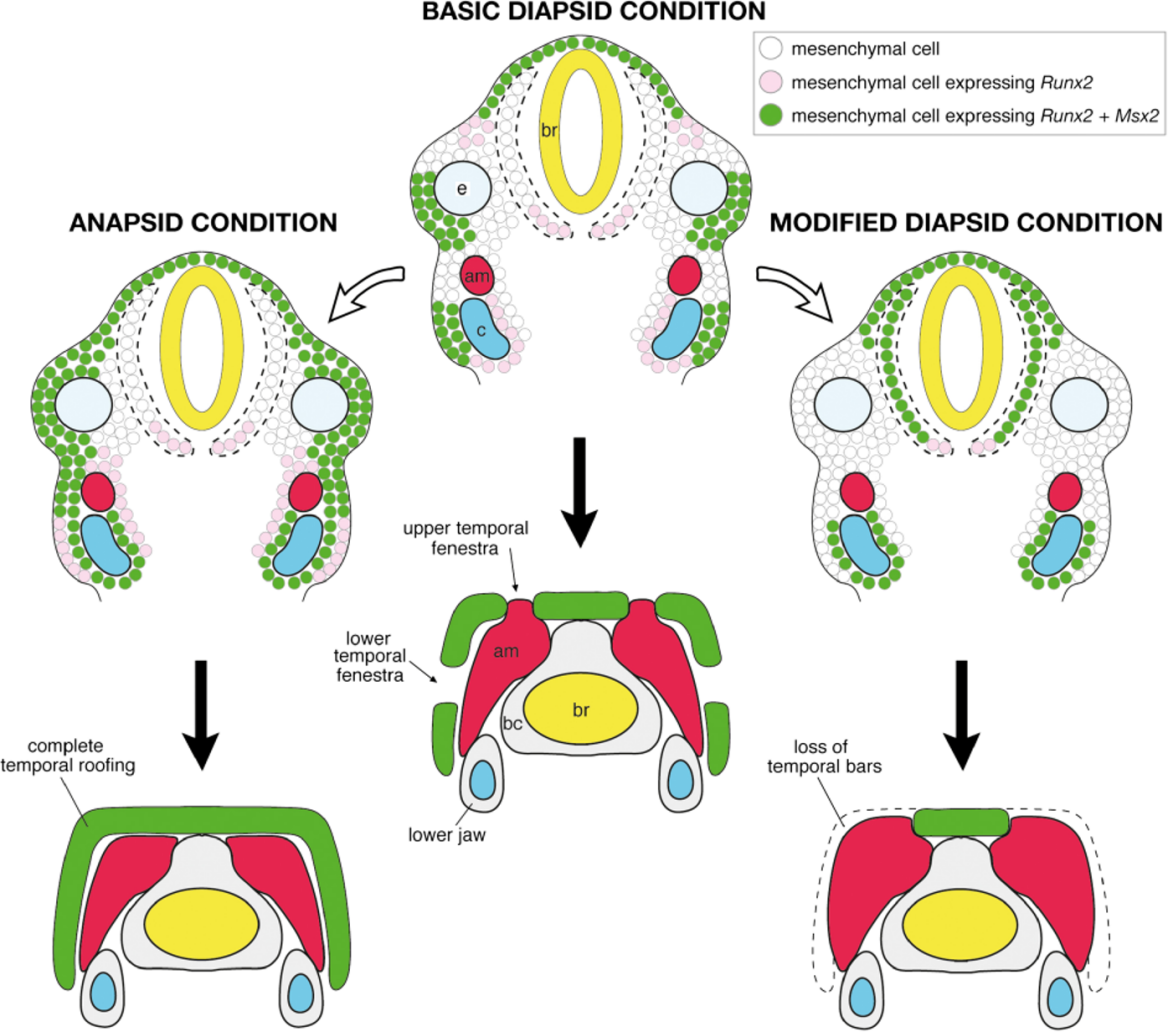
**Potential developmental basis that generates morphological diversity in the temporal region of the reptilian skull.** All extant reptilian lineages are considered to be derived from ancestor with diapsid skull. In crocodiles that have both upper and lower temporal bars like the stem Diapsida (e.g., *Petrolacosaurus*), osteogenic mesenchymal precursor cells which express *Runx2* and/or *Msx2* are distributed at the domain where future temporal bars are formed in the head of early stage embryo (top of the middle column). Through ontogeny (black arrow), these osteoblast precursors may differentiate into the dermatocranial elements including upper and lower temporal bars (bottom of the middle column). Between these bony bars, both upper and lower temporal fenestrae are clearly recognized. In turtles (left white arrow), distribution of osteogenic mesenchymal precursor cells is broadened in a dorsal-ventral direction, filling the whole lateral portion of the head of early embryos (top of the left column). Through ontogeny, these osteoblast precursors may differentiate into the dermatocranial elements roofing the temporal region of the head (bottom of the left column). In snakes that have modified diapsid skull where temporal bars are absent, osteogenic mesenchymal precursor cells do not fill lateral domain of the head of embryo. Rather, these cells are mainly distributed in the vicinity of the brain (top of the right column). Through ontogeny, these osteoblast precursors may differentiate into the dermatocranial elements accompanying the braincase, without forming bony temporal bars (bottom of the right column). A condensed mesenchymal layer that differentiates into the braincase in later stages is highlighted by dotted line in the head of embryos.

Runx2 is widely known as a transcription factor that plays a fundamental role in osteoblast differentiation in vertebrate embryos (Ducy et al., 1997, Komori et al., 1997, Mundlos et al., 1997, Nakashima et al., 2002, Ishii et al., 2003, Dobreva et al., 2006, Kerney et al., 2010) and its transcript has been used as a molecular marker for preosteoblasts or osteoblast progenitors (Ducy et al., 1997, Karsenty, 2001, Bobola et al., 2003, Ishii et al., 2003, Abzhanov et al., 2007, Han et al., 2007;Tokita & Schneider , Tokita & Schneider Tokita & Schneider 2009). In turtle embryos where mineralization of dermal bones of the skull has not yet occurred, mesenchymal cells that express *Runx2* were broadly distributed in the lateral domain of the head, from the top of the head to the ventral margin of the jaw (Figure 9). This means that turtle embryos have wider distribution of the cells that have a potential to differentiate into bones in the temporal region of the head, compared to other lineages of reptiles. The reduction of the amount of *Runx2* mRNA causes developmental defects in calvarial bones, called cleidocranial dysplasia, in mouse embryos (Lou et al., 2009). In contrast, early onset of *Runx2* expression that eventually results in an increase of the amount of its mRNA in the cranial mesenchyme accelerates the timing of mineralization of cranial dermal bones in mouse embryos and brings about craniosynostosis characterized by overgrowth of bones (Maeno et al., 2011). Similar result has been obtained from experiments using avian embryos (Merrill et al., 2008). We speculate that heterotopy in the preosteoblast distribution observed in early stage turtle embryos may lead to the increase of the amount of *Runx2* expression that results in the increase of the level of ossification in the temporal region of their skull and this would be a primary factor to build the anapsid skull where the temporal region is completely covered with bone.

In this study, we focused on several candidate molecules that potentially regulate *Runx2* expression and examined their expression patterns in reptilian embryos. Bmp4 is known to be involved in osteogenesis of vertebrates where it regulates expression of other osteogenic regulatory genes, including *Msx1*, *Msx2*, and *Runx2* (Marazzi et al., 1997, Kim et al., 1998, Hollnagel et al., 1999, Tribulo et al., 2003, Zhang et al., 2003, Brugger et al., 2004). Msx1 is a transcription factor known to regulate growth and patterning of calvarial bones in mouse embryos (Satokata & Maas, 1994, Han et al., 2007, Roybal et al., 2010). Although, as previously reported in mouse embryos (Rice et al., 2003, Han et al., 2007), both *Bmp4* and *Msx1* are expressed in limited populations of cranial mesenchyme in embryos of crocodiles and turtles, we could not detect any substantial differences in their expression domains between the two species. On the other hand, we observed spatially different expression patterns of *Msx2* in the head of embryos of all reptilian species we examined. Expression of *Msx2* was detected in cranial mesenchyme and dermal bone precursors as reported in mouse embryos (Jabs et al., 1993, Ishii et al., 2003, Rice et al., 2003, Han et al., 2007, Roybal et al., 2010). Furthermore, its expression spatially overlapped with that of *Runx2* in reptilian embryos, as in mouse embryos (Ishii et al., 2003, Rice et al., 2003, Han et al., 2007). In turtle embryos, expression domain of *Msx2* in the mesenchyme distributed in the temporal region of the head was broad in a dorsal-ventral direction, showing similar pattern with *Runx2* in the mesenchyme. A mutation in the homeobox of *Msx2* gene causes craniosynostosis in human and mouse (Jabs et al., 1993, Liu et al., 1999). Similarly, overexpression of *Msx2* promotes osteogenesis (Cheng et al., 2003, Ichida et al., 2004) and causes overgrowth of dermal bones of the skull by increasing the number of proliferative osteoblasts (Dodig et al., 1999, Liu et al., 1999). In contrast, loss-of-function of *Msx2* results in defects of skull ossification in mammals (Satokata et al., 2000, Wilkie et al., 2000, Ishii et al., 2003, Antonopoulou et al., 2004, Han et al., 2007). Furthermore, Msx2 is known to positively regulate downstream *Runx2* expression (Ishii et al., 2003, Han et al., 2007, Watanabe et al., 2008). Considering the evidence provided by previous studies, regulatory changes in *Msx2* expression in turtle embryos may influence expression patterns of downstream *Runx2*, which regulate osteoblast differentiation. Dorsoventrally broadened distribution of osteogenic mesenchymal precursor cells in the temporal region of the head owing to the regulatory alteration of these osteogenic genes may allow this reptilian lineage to reacquire the anapsid skull. Although the precise mechanism underlying regulatory change of *Msx2* expression in the head of turtle embryos has not been identified, recent findings that early stage arrest of *Msx2* expression in neural crest-derived odontoblasts may account for the absence of teeth in turtles (Tokita et al., 2012) supports the hypothesis that this transcription factor may play a pivotal role in the development of their unique cranial morphology.

The development of the dermatocranium occurs in multiple steps (Ishii et al., 2003). The first phase includes the genesis, migration, and initial specification of osteogenic mesenchymal precursor cells. The second phase consists of the differentiation of the mesenchyme into osteoblasts. And the last phase includes deposition of osteogenic extracellular matrix around the osteoblasts and mineralization of the matrix. The dermatocranium of vertebrates is formed from cranial mesenchyme derived from two distinct embryonic sources: neural crest and mesoderm (Jiang et al., 2002;Gross & Hanken , Gross & Hanken Gross & Hanken 2005;Noden & Trainor,, Noden & Trainor, Noden & Trainor,^2005^). Unfortunately, fate mapping studies of each dermatocranial element as performed in avian and mammalian embryos (Le Lièvre, 1978, Noden, 1978,1983, Couly et al., 1993;Köntges & Lumsden,, Köntges & Lumsden, Köntges & Lumsden,^1996^, Jiang et al., 2002) have not been done in non-avian reptiles. Interestingly, the pattern of migration and distribution of cranial neural crest cells from which some cranial dermal bones should form is almost identical in early stage embryos of crocodiles and turtles (Meier & Packard, 1984;Hou & Takeuchi,, Hou & Takeuchi, Hou & Takeuchi,^1994^;Kundrát,, Kundrát, Kundrát,^2008^). Such data may support that differentiation or maturation processes of osteogenic mesenchyme are more responsible for producing diversity of reptilian skull morphology. We speculate that the developmental program, which determines cranial mesenchymal populations where early-phase osteogenic transcription factors Msx2 and Runx2 are activated, may be important in the patterning of reptilian skull morphology.

There exists substantial diversity in the skull morphology within turtles and most living turtle species do not have fully anapsid skulls and instead possess varying degrees of dorsal and/or ventral emargination on their skull (Jones et al., 2012, Werneburg, 2012a). In the present study, we could not sample and analyze the embryos of turtle species with fully anapsid skull, such as marine turtles (Kuratani, 1999, Jones et al., 2012), alligator snapping turtle () (Sheil, *Macrochelys temminckii*^2005^), and big-headed turtle (*Platysternon megacephalum*), owing to difficulty in the access to the materials. Instead, we analyzed the embryos of a soft-shelled turtle species with highly emarginated skull. In fact, soft-shelled turtles have only a narrow bar of bone across the temporal region lateral to the external adductor muscles due to large scale emargination from the dorsal and ventral margins of the cheek (Ogushi, 1911, Sheil, 2003). In normal development of soft-shelled turtles, the postorbital bone does not grow in a posterior direction significantly, keeping its relatively small size within the dermatocranium (Sheil, 2003;Sánchez-Villagra et al.,, Sánchez-Villagra et al., Sánchez-Villagra et al.,^2009^). Therefore, the small postorbital bone of soft-shelled turtles does not largely contribute to the formation of a bony roof at the temporal region of the skull.

It is interesting that we observed dorsoventrally broadened distribution of the mesenchymal cells that express *Runx2* at the temporal region of the embryos of a soft-shelled turtle species with highly emarginated skull. Dermal bone development occurs through a multi-step molecular pathway regulated by different transcription factors (Zhang, 2010). As an initial step, *Runx2* is required for the differentiation of mesenchymal cells into preosteoblasts. In subsequent stage where these preosteoblasts differentiate into mature osteoblasts, *Osx*, a downstream gene of *Runx2*, is necessary (Nakashima et al., 2002, Nishio et al., 2006). Furthermore, in the later stages where the osteoblasts produce osteogenic extracellular matrix and the mineralization of these extracellular matrix is occurred, many additional molecules such as bone sialoprotein, osteopontin, and osteocalcin are involved (Zhang, 2010). We speculate that in soft-shelled turtles only a limited population of cells within *Runx2*-positive preosteoblasts distributed in the temporal region of the head is allowed to differentiate into mature osteoblasts and eventually osteocytes through regulation of expression of down stream genes (e.g. *Osx*), to form a pair of relatively small postorbital bones. Although the regulatory mechanism of *Osx* expression in osteogenic mesenchyme is not fully understood, both Runx2-dependent and -independent pathways have been suggested (Lee et al., 2003;Celil & Campbell,, Celil & Campbell, Celil & Campbell,^2005^, Maehata et al., 2006, Xing et al., 2007, Zhang, 2010). Histological analysis reveals that late stage soft-shelled turtle embryos have a layer of (non-muscular) fibrous connective tissue lateral to the external adductor muscles (Additional file 3). Judging from its position, the connective tissue layer appears to be derived from *Runx2*-positive preosteoblasts and have a potential to ossify themselves as other connective tissues represented by tendons and ligaments (Okawa et al., 1998;Tokita et al., 2007). Interestingly, similar type of connective tissue layer is absent in the temporal region of crocodile and snake embryos (Additional file 3). Those histological observations support the above hypothesis that later processes of cranial osteogenesis may largely contribute to the construction of the main body of each dermatocranial element from the osteogenic mesenchymal progenitor pool.

The dorsoventrally broadened distribution of preosteoblasts observed in turtle embryos might be a developmental synapomorphy re-acquired by the common ancestor of turtles. In the course of chelonian evolution, each chelonian lineage may develop the temporal dermal bones (e.g. postorbital, parietal, jugal) with various sizes and shapes, through regulatory changes of the osteogenic down stream molecules. Future studies should investigate expression pattern of *Runx2* and *Msx2* in the head of embryos of turtle species with fully anapsid skull, as well as expression pattern of downstream genes that regulate differentiation of mature osteoblasts and osteocytes in turtle embryos, to verify a correlation between the gene expression pattern and their skull morphology.

### Heterotopy in distribution of osteogenic mesenchymal precursor cells and diversification of reptilian skull morphology

The frame-like skulls possessed by diapsid reptiles evolved in response to functional forces (Rieppel, 1993a, Moazen et al., 2009, Herrel et al., 2007, Curtis et al., 2011) and several studies have suggested heterochrony as a driving force for producing this morphological diversity (Rieppel, 1993a, Whiteside, 1986, Irish, 1989). The ancestral lineage of diapsid reptiles possessed upper and lower temporal bars that encircle temporal fenestrae (Müller, 2003, Moazen et al., 2009). The lower temporal bar that encloses lower temporal fenestra ventrally was probably lost once in the common ancestor of lepidosaurs and archosaurs, possibly as the outcome of paedomorphosis: incomplete ossification of a quadrato-maxillary ligament between jugal and quadratojugal bones (Rieppel, 1993a;Müller,, Müller, Müller,^2003^). If this is true, the lower temporal bar that possibly results from peramorphosis (hypermorphosis): complete ossification of a quadrato-maxillary ligament was independently re-acquired in the lineages of tuatara and crocodiles, as well as in several extinct reptilian lineages (Rieppel, 1993a;Müller,, Müller, Müller,^2003^). Furthermore, disappearance of upper temporal bar, which is regarded as an extreme condition of reduction of the dermatocranium in reptiles, may have independently evolved in the skull of geckos (Gekkonidae), miniaturized fossorial lizards (e.g., *Typhlosaurus*, *Dibamus*), amphisbaenian, and snakes, as the outcome of paedomorphosis represented by the retardation of ossification (Rieppel, 1993a, Irish, 1989;Cundall & Irish,, Cundall & Irish, Cundall & Irish,^2008^). In the present study, we revealed a possible correlation between distribution pattern of *Runx2* and/or *Msx2*-expressing osteogenic mesenchymal precursor cells and the skull morphology of each reptilian lineage (Figure 9). In early stage crocodile embryos, we observed focal distribution of osteogenic mesenchyme around the domain where future temporal bars are formed. In early stage snake embryos, osteogenic mesenchymal cells were primarily found adjacent to the primordium of the braincase and the spatial pattern presaged the absence of bony temporal bars in the temporal region of adult animal.

## Conclusions

Regulatory modifications of *Runx2* and *Msx2* expression in osteogenic mesenchymal precursor cells are likely involved in generating morphological diversity in the temporal region of the reptilian skull, including secondary closure of the temporal fenestrae in turtles. Our findings demonstrate that not only heterochrony in ossification of the dermatocranium that has been traditionally regarded as the major factor producing diversity of reptilian cranial morphology but also heterotopy in distribution of the osteogenic precursor cells may play a fundamental role in this process and it should be further investigated in future studies of reptilian cranial development and evolution.

## Materials and methods

### Sample collection and staging of embryos

Fertilized eggs of Chinese soft-shelled turtle, *Pelodiscus sinensis*, were purchased commercially from a local breeder in Japan. Fertilized eggs of Siamese crocodile, *Crocodylus siamensis*, were provided by a local breeder in Thailand. Fertilized eggs of corn snake, *Pantherophis guttatus*, were obtained by the first author after mating several pairs of the reproductively mature adults in the laboratory. Staging of *P*. *sinensis* embryos was performed after Tokita and Kuratani (2001). Because there is no embryonic staging system for *C*. *siamensis* at present, we used the system for *Alligator mississippiensis* embryos (Ferguson, 1985) where each stage was determined based on external morphology of the embryos, for staging of this species. Staging of *P*. *guttatus* (Zehr, embryos was performed on the basis of staging table of *Thamnophis sirtalis*^1962^). Interspecific comparisons of gene expression pattern were performed in the embryos that are comparable to each other in terms of overall external morphology. Because snake embryos are limbless, we mainly employed external features of the head of the embryos as primary criteria for determining the stages for comparison. All animal experiments were approved by the University of Tsukuba Committee for Animal Care (No.10-034).

### Molecular cloning

Total RNA was extracted from embryos using ISOGEN reagent (NIPPON GENE CO., LTD).

RT–PCR was performed to amplify fragments of *P*. *sinensis Runx2*, *Six2* and *C. siamensis Bmp4*, *Msx2*, *MyoD*, *Runx2*, *Scleraxis* (*Scx*), *Six2*, *Sox9* and *P*. *guttatus Msx2*, *Runx2*, *Sox9* messenger RNA. Primer sequences used for isolation of the fragments of these genes are available upon request. Because *Bmp4*, *Msx1*, *Msx2*, *MyoD*, *Scx*, *Sox9* of *Pelodiscus* and *MyoD* of *Pantherophis* were already sequenced and sequence data were deposited in the database by other researchers, we isolated the orthologous fragments by RT–PCR with primers constructed by referring to the reported sequence data. The fragments were isolated using the pGEM T-easy vector systems (Promega) or TOPO® TA cloning kit (Invitrogen) and sequenced using an ABI 3130 sequencer (Applied Biosystems). To identify the orthologous genes of the isolated fragments, comparable sequence data were surveyed using a BLAST search, and phylogenetic trees with neighbor joining method were constructed after sequence alignment using the CLUSTALX software. All new DNA sequence data were deposited in the DDBJ database (AB811933-AB811944).

### Gene expression analysis

Embryos were fixed in 4% PFA, dehydrated using an methanol series, placed in xylene, embedded in paraffin, and sliced with a microtome. Serial sections were hybridized with digoxigenin-labeled RNA riboprobes as described in Neubüser et al. (1995) with slight modifications. To identify the expression domain of *Msx1* in crocodile tissues, chicken *Msx1* antisense riboprobe was hybridized. Generally, hetero-specific RNA probes easily hybridize among reptilian lineages (Harris et al., 2006, Tokita et al., 2012). In this study, we only analyzed reptilian embryos at the ontogenetic stages where early cranial osteogenesis occurs. To confirm the expression pattern of each gene in the cranial tissues, two to five individuals representing each embryonic stage were sampled for analysis. The consistency of the gene expression patterns among all individual embryos at the same stage was confirmed. Multiple sections representing several longitudinal (anterior-posterior) planes prepared from the same individual were hybridized with the probes and the sections prepared at corresponding longitudinal planes were compared between different individuals. Corresponding longitudinal planes between different reptilian species were determined based on overall histological configuration of the head of the embryos. For visualization of each cranial tissue and interspecific comparison of general histology of the head, Miligan's Trichrome staining was performed following standard protocols. To identify each anatomical structure in cranial musculoskeletal tissues of the embryos, we took the results of other's researches into account: (Schumacher, 1973, Rieppel, 1993b;Vickaryous & Hall,, Vickaryous & Hall, Vickaryous & Hall,^2008^;Bona & Desojo,, Bona & Desojo, Bona & Desojo,^2011^) for crocodile, (Schumacher, 1973, Rieppel, 1990, Rieppel, 1993c;Sánchez-Villagra et al.,, Sánchez-Villagra et al., Sánchez-Villagra et al.,^2009^, Werneburg, [Bibr CR127][Bibr CR128]) for turtle, and (Kamal et al., 1970, Haas, 1973, Zaher, 1994;Buchtová et al.,, Buchtová et al., Buchtová et al.,^2007^) for snake.

## Electronic supplementary material

Additional file 1: **Expression of musculoskeletal tissue marker genes in the head of crocodile embryos at stage 14.** (A) Lateral view of the embryo. (B-D, and F) Frontal sections prepared around the plane indicated by the blue line in (A). (E) Frontal section prepared around the plane indicated by the red line in (A). (B) Expression of *Runx2* is faintly detected at the mesenchymal cells above and below the eye, as well as in the mesenchyme distributed medial to the precursor of quadrate cartilage and in the mesenchyme surrounding the braincase (arrows). (C) Cranial muscular tissues are clearly labeled by *MyoD* probe. (D) Cartilaginous tissues, including the braincase and the quadrate (q), are labeled by *Sox9* probe. (E) *Scx* is expressed in tendon precursor cells in close proximity of *MyoD*-positive jaw and eye muscle anlagen (arrowheads). (F) *Six2* is expressed mainly in the mesenchyme around *Sox9*-positive cartilage precursors, including the quadrate and the braincase, as well as in the mesenchyme around *MyoD*-positive cranial muscle anlagen. The red outlined domains indicate the location of the anlagen of the jaw muscle complex. Scale bar in (A) is 1 mm. Scale bars in (B-F) are 0.5 mm. (TIFF 9 MB)

Additional file 1: **Expression of musculoskeletal tissue marker genes in the head of turtle embryos at stage 14.** (A) Lateral view of the embryo. (B-E) Frontal sections prepared around the plane indicated by the red line in (A). (B) *Runx2*-positive mesenchymal cells are distributed above and below the eye, as well as in the domain medial to the anlagen of jaw adductor muscle (black arrows). (C) Cranial muscular tissues are clearly labeled by *MyoD* probe. (D) Cartilaginous tissues, including the braincase and quadrate, are labeled by *Sox9* probe. (E) *Six2* is expressed mainly in the mesenchyme around *Sox9*-positive cartilage precursors and *MyoD*-positive cranial muscle anlagen. Scale bar in (A) is 1 mm. Scale bars in (B-E) are 0.5 mm. (TIFF 5 MB)

Additional file 1: **A layer of fibrous connective tissue lateral to the external adductor muscle is found in late stage soft-shelled turtle embryo.** (A) Lateral view of the head of a turtle embryo at stage 22. (B, C) Frontal sections of the head prepared in the planes indicated in (A). Note a clear layer of fibrous connective tissue lateral to the external adductor muscle (red arrowheads). (D) Lateral view of the head of a crocodile embryo at stage 20. (E, F) Frontal sections of the head prepared in the planes indicated in (D). (G) Lateral view of the head of a snake embryo at stage 31. (H, I) Frontal sections of the head prepared in the planes indicated in (G). A layer of fibrous connective tissue is not seen in the domain lateral to the external adductor muscle in crocodile and snake embryos. Rather, in crocodile and snake embryos, the domain is occupied by mesenchymal cells in low density or by acellular cavities. Scale bars are 1 mm. (TIFF 6 MB)

## Abbreviations

Am: Jaw adductor muscle or the anlage of jaw adductor muscle
Ame: External adductor muscle
Bc: Braincase
Bp: Basicranial plate cartilage
Br: Brain
C: precursor of jaw cartilages (quadrate and Meckel's)
Co: Cochlear canal
E: Eye
Em: Eye muscles or the anlagen of eye muscles
Itm: Intramandibular muscle
J: Jugal bone
Jm: Anlage of jaw muscle complex
Mc: Meckel's cartilage
Mx: Primordium of maxilla bone
Oc: Oral cavity
P: Parietal bone or precursor of parietal bone
Pc: Parachordal cartilage
Pg: Primordium of pterygoid bone
Pgm: Pterygoid muscle
Pl: Primordium of palatine bone
Po: Postorbital bone or primordium of postorbital bone
Ptm: Pseudotemporal muscle
Q: Quadrate cartilage or precursor of quadrate cartilage
Qj: Quadratojugal bone or primordium of quadratojugal bone
T: Trabecular cartilage
Tr: Ganglion of trigeminal nerve
1a: First pharyngeal arch
2a: Second pharyngeal arch
