# Supplementary figures and images for: Skeletal gene expression in the temporal region of the reptilian embryos: implications for the evolution of reptilian skull morphology
Source: Springerplus. 2013 Jul 23;2:336. doi: 10.1186/2193-1801-2-336 (PMC3970585; doi:10.1186/2193-1801-2-336)

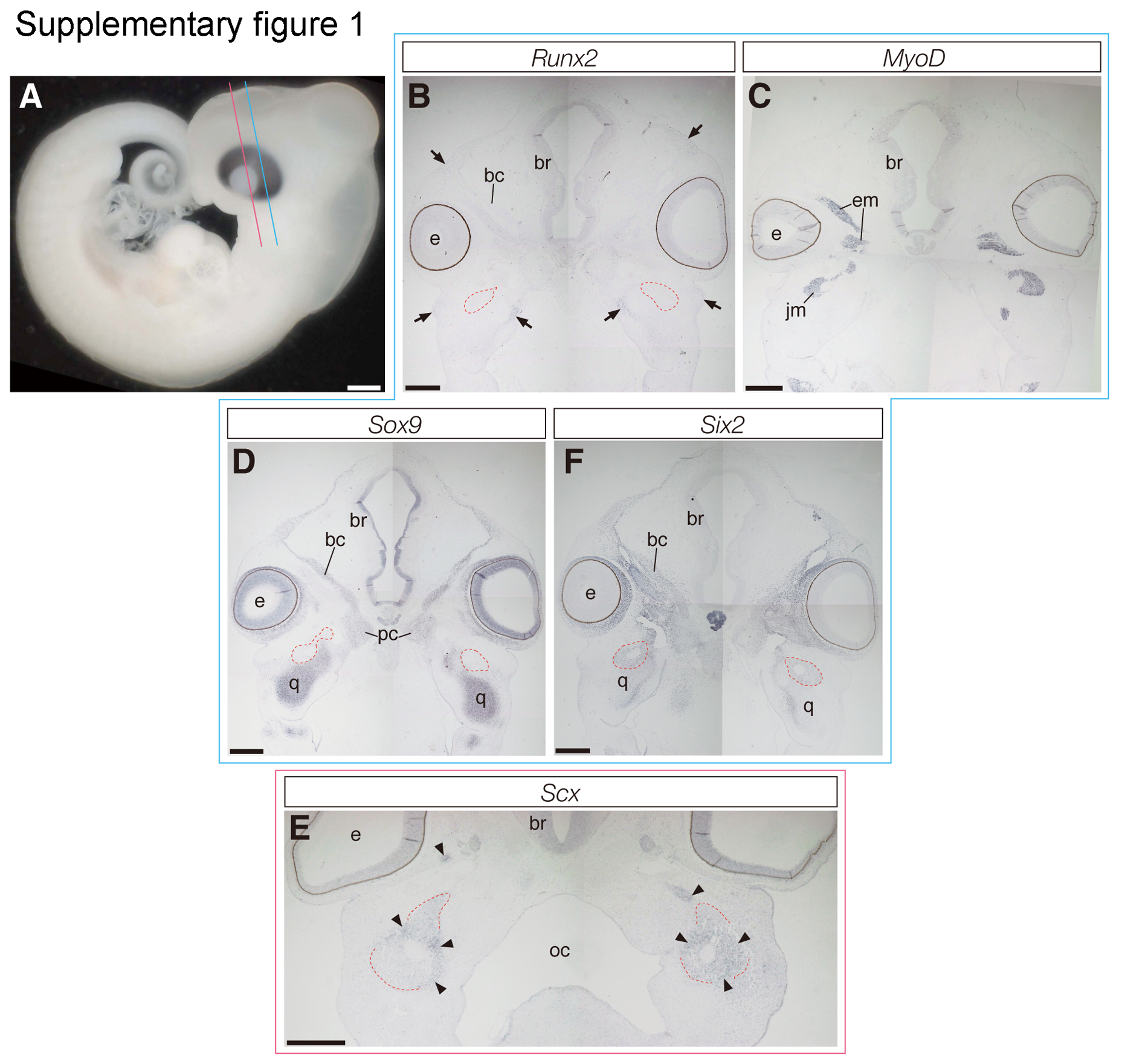

Supplement: Supplementary file 1 — Additional file 1: Expression of musculoskeletal tissue marker genes in the head of crocodile embryos at stage 14. (A) Lateral view of the embryo. (B-D, and F) Frontal sections prepared around the plane indicated by the blue line in (A). (E) Frontal section prepared around the plane indicated by the red line in (A). (B) Expression of Runx2 is faintly detected at the mesenchymal cells above and below the eye, as well as in the mesenchyme distributed medial to the precursor of quadrate cartilage and in the mesenchyme surrounding the braincase (arrows). (C) Cranial muscular tissues are clearly labeled by MyoD probe. (D) Cartilaginous tissues, including the braincase and the quadrate (q), are labeled by Sox9 probe. (E) Scx is expressed in tendon precursor cells in close proximity of MyoD-positive jaw and eye muscle anlagen (arrowheads). (F) Six2 is expressed mainly in the mesenchyme around Sox9-positive cartilage precursors, including the quadrate and the braincase, as well as in the mesenchyme around MyoD-positive cranial muscle anlagen. The red outlined domains indicate the location of the anlagen of the jaw muscle complex. Scale bar in (A) is 1 mm. Scale bars in (B-F) are 0.5 mm. (TIFF 9 MB) [file 40064_2013_438_MOESM1_ESM.tiff]

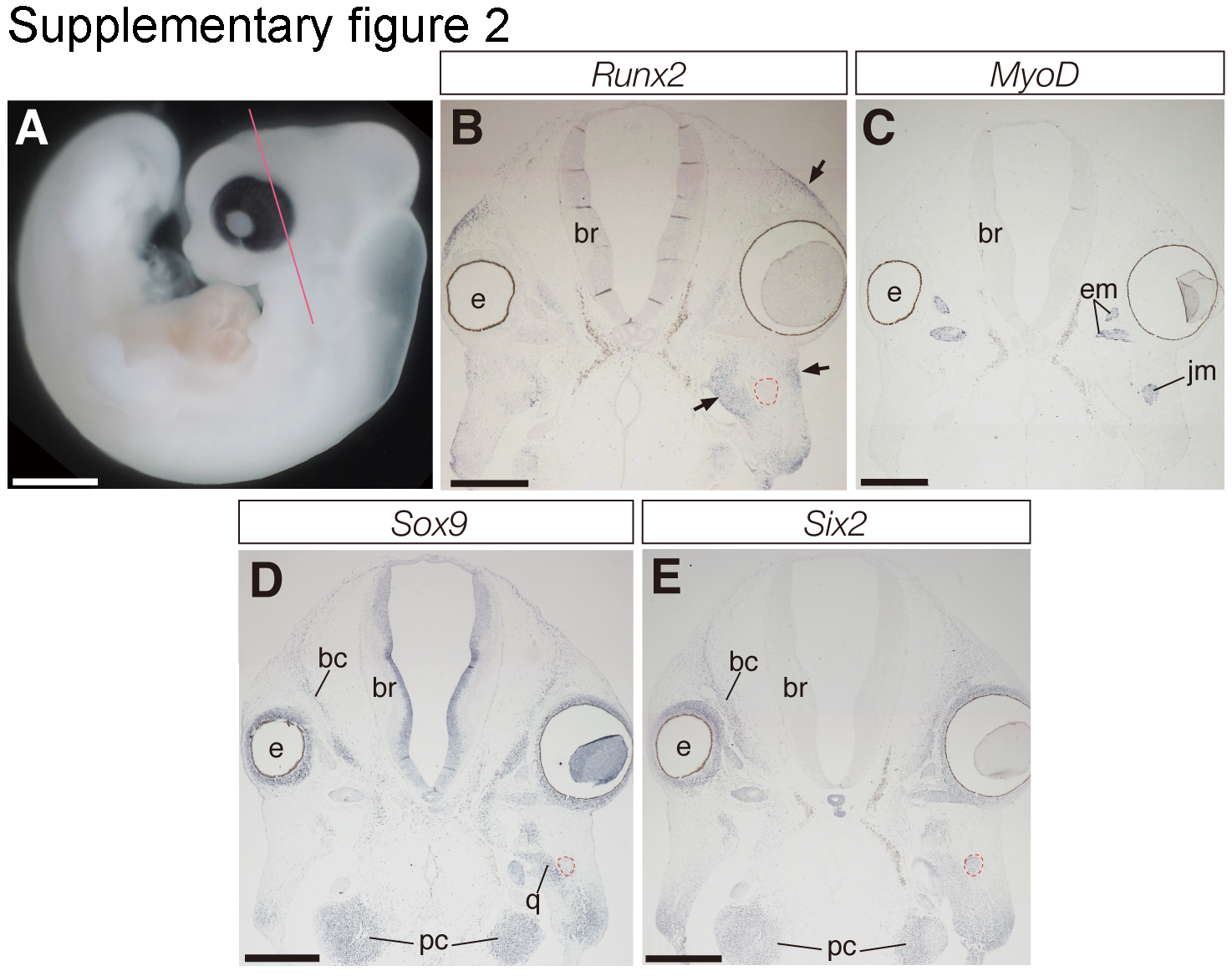

Supplement: Supplementary file 2 — Additional file 1: Expression of musculoskeletal tissue marker genes in the head of turtle embryos at stage 14. (A) Lateral view of the embryo. (B-E) Frontal sections prepared around the plane indicated by the red line in (A). (B) Runx2-positive mesenchymal cells are distributed above and below the eye, as well as in the domain medial to the anlagen of jaw adductor muscle (black arrows). (C) Cranial muscular tissues are clearly labeled by MyoD probe. (D) Cartilaginous tissues, including the braincase and quadrate, are labeled by Sox9 probe. (E) Six2 is expressed mainly in the mesenchyme around Sox9-positive cartilage precursors and MyoD-positive cranial muscle anlagen. Scale bar in (A) is 1 mm. Scale bars in (B-E) are 0.5 mm. (TIFF 5 MB) [file 40064_2013_438_MOESM2_ESM.tiff]

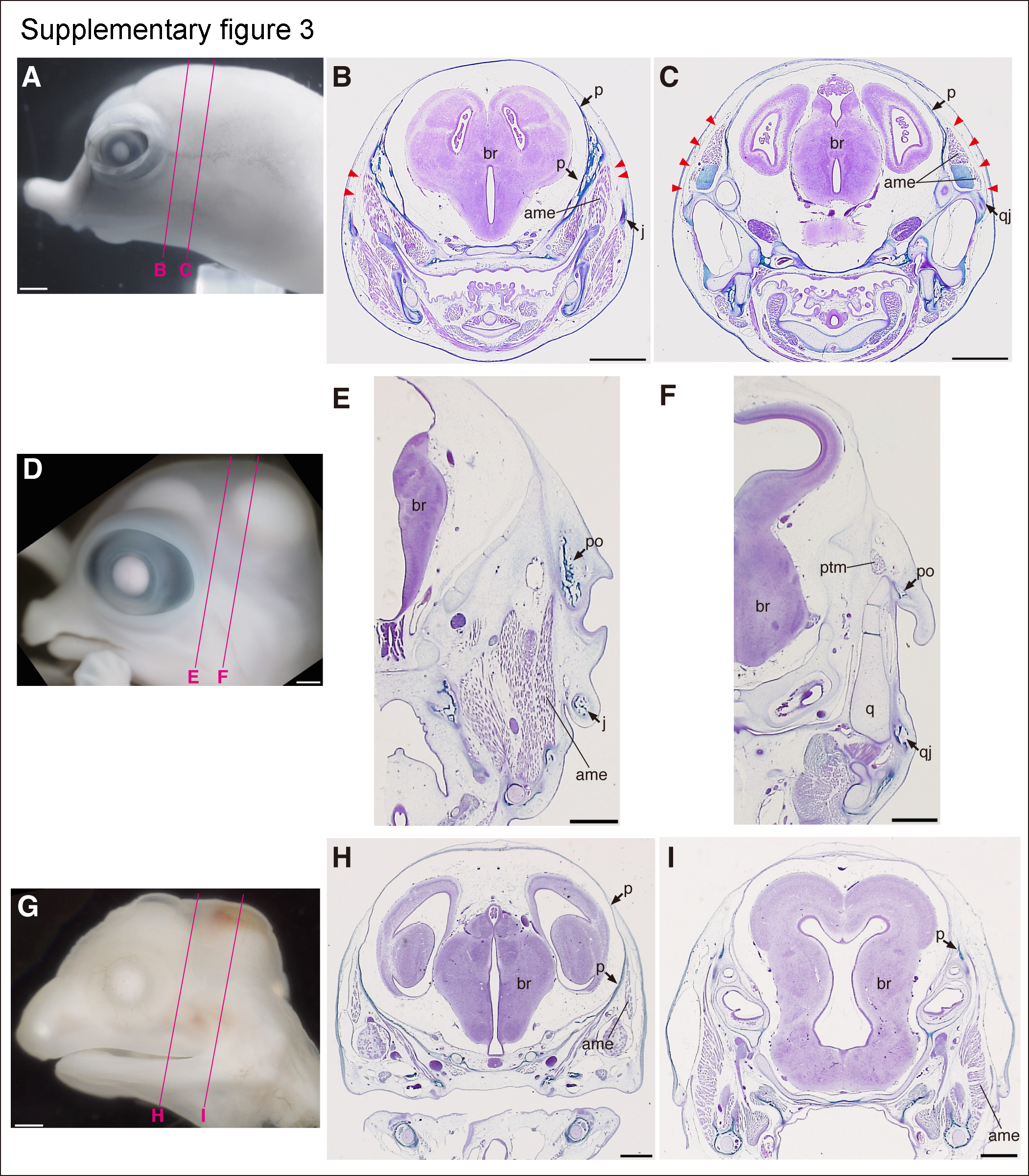

Supplement: Supplementary file 3 — Additional file 1: A layer of fibrous connective tissue lateral to the external adductor muscle is found in late stage soft-shelled turtle embryo. (A) Lateral view of the head of a turtle embryo at stage 22. (B, C) Frontal sections of the head prepared in the planes indicated in (A). Note a clear layer of fibrous connective tissue lateral to the external adductor muscle (red arrowheads). (D) Lateral view of the head of a crocodile embryo at stage 20. (E, F) Frontal sections of the head prepared in the planes indicated in (D). (G) Lateral view of the head of a snake embryo at stage 31. (H, I) Frontal sections of the head prepared in the planes indicated in (G). A layer of fibrous connective tissue is not seen in the domain lateral to the external adductor muscle in crocodile and snake embryos. Rather, in crocodile and snake embryos, the domain is occupied by mesenchymal cells in low density or by acellular cavities. Scale bars are 1 mm. (TIFF 6 MB) [file 40064_2013_438_MOESM3_ESM.tiff]
